# DEEP-squared: deep learning powered De-scattering with Excitation Patterning

**DOI:** 10.1038/s41377-023-01248-6

**Published:** 2023-09-13

**Authors:** Navodini Wijethilake, Mithunjha Anandakumar, Cheng Zheng, Peter T. C. So, Murat Yildirim, Dushan N. Wadduwage

**Affiliations:** 1 Center for Advanced Imaging, Faculty of Arts and Sciences, Harvard University, Cambridge, MA USA; 2 Department of Electronic and Telecommunication Engineering, University of Moratuwa, Moratuwa, Sri Lanka; 3 Department of Mechanical Engineering, Massachusetts Institute of Technology, 77 Massachusetts Ave., Cambridge, MA 02139 USA; 4 Laser Biomedical Research Center, Massachusetts Institute of Technology, 77 Massachusetts Ave., Cambridge, MA 02139 USA; 5 Department of Biological Engineering, Massachusetts Institute of Technology, 77 Massachusetts Ave., Cambridge, MA 02139 USA; 6 Picower Institute for Learning and Memory, Massachusetts Institute of Technology, 77 Massachusetts Ave., Cambridge, MA 02139 USA; 7 Department of Neuroscience, Cleveland Clinic Lerner Research Institute, Cleveland, OH 44195 USA

**Keywords:** Multiphoton microscopy, Imaging and sensing

## Abstract

Limited throughput is a key challenge in in vivo deep tissue imaging using nonlinear optical microscopy. Point scanning multiphoton microscopy, the current gold standard, is slow especially compared to the widefield imaging modalities used for optically cleared or thin specimens. We recently introduced “De-scattering with Excitation Patterning” or “DEEP” as a widefield alternative to point-scanning geometries. Using patterned multiphoton excitation, DEEP encodes spatial information inside tissue before scattering. However, to de-scatter at typical depths, hundreds of such patterned excitations were needed. In this work, we present DEEP^2^, a deep learning-based model that can de-scatter images from just tens of patterned excitations instead of hundreds. Consequently, we improve DEEP’s throughput by almost an order of magnitude. We demonstrate our method in multiple numerical and experimental imaging studies, including in vivo cortical vasculature imaging up to 4 scattering lengths deep in live mice.

## Abstract (web summary)

We present DEEP-squared, a machine learning model trained physics-informed to improve widefield two-photon imaging speed by nearly an order of magnitude for de-scattering with excitation pattering (DEEP).

## Graphical abstract

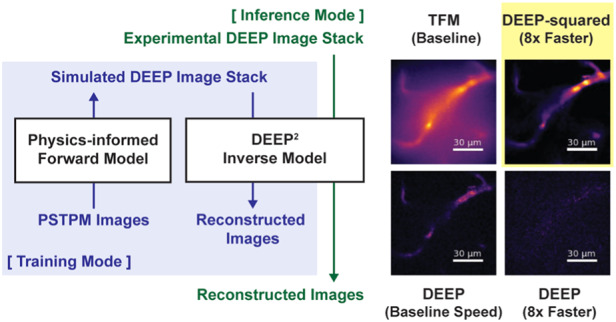

## Introduction

Imaging biological structures such as neurons or blood vessels deep inside scattering tissue is an important yet challenging microscopy problem. For such in vivo deep tissue experiments, today, the gold standard is the point-scanning two- (or three-) photon microscopy (PSTPM)^1–3^. Especially, PSTPM occupies a unique position in neuroscience due to its high spatial resolution, low phototoxicity, and ability to penetrate through deep tissue. In operation, PSTPM focuses femtosecond laser light at long wavelengths through scattering tissue to excite fluorescent molecules. Due to long wavelengths, the excitation light sees only slight scattering. The microscope then collects emission fluorescence through the same scattering tissue onto a detector. This emission light, however, is at a much shorter wavelength and hence encounters significant scattering on its way to the detector. For this reason, multiple points cannot be excited and resolved simultaneously. So imaging is performed sequentially, one point at a time. Thus, PSTPM is inherently a slow imaging technique. When speed is needed, either resolution or the imaging field of view should be compromised^4^.

Despite the limitations due to emission light scattering, widefield two- and three-photon microscopes (WFTPM) have been proposed. For instance, temporal focusing microscopy (TFM) focuses amplified femtosecond laser pulses temporally for depth-selective WFTPM. TFM provides excellent optical sectioning and also penetrates well through scattering tissue due to long multiphoton excitation wavelengths^5–8^. Yet, the emission light scatters. Therefore, in widefield deep tissue imaging, some photons are mapped onto incorrect detector pixels, degrading the signal-to-noise ratio and spatial resolution. A few groups, including us, recently proposed to overcome this limitation by combining TFM with structured light illumination. Since excitation light penetrates through the scattering medium, structured illuminations can modulate the imaging field of view before scattering. Thus, the information about the structured illuminations can be used to de-scatter TFM images despite degraded detection. Using this principle, Escobet et al.^9^ proposed “TempoRAl Focusing microscopy with single-pIXel detection (TRAFIX).” They used coded excitations and single-pixel detection. Subsequently, we proposed “De-scattering with Excitation Patterning (DEEP)”^10^. Instead of single-pixel detection, we used widefield detection. Single-pixel detection completely relies on coded illuminations for de-scattering. Widefield detection, however, retains some spatial information and only partially depends on coded illumination. Thus, while TRAFIX needs tens of thousands of illumination patterns to reconstruct a typical WFTPM image, DEEP requires only hundreds. Despite such speed improvement, DEEP is still slower than other depth-resolved widefield imaging modalities used for optically cleared or thin specimens. For instance, one-photon fluorescence modalities, that optically section with planar or structured illumination, are either single-shot or few-shot acquisition^11^. Therefore, in this work, we attempt to further increase the throughput of DEEP by utilizing prior image information within a deep-learning-based computational imaging framework.

From a computational imaging point of view, the imaging process that describes the translation of the ideal image (*x*) to the observed image (*y*) is called the forward model (*f*). During the forward imaging process, the observed image (*y*) is degraded by image noise; low-pass filtering; pixel-value quantization; sub-sampling; and, in our case, scattering^12^. An inverse model is needed to map the observed (*y*) back to the ideal expected image (*x*). In our original work^10^, we used an analytical inverse model with wavelet sparsity priors. However, recently, for such inverse problems, deep learning has proven impressively capable^12–15^. For example, deep learning has shown outstanding success in image classification^16,17^, biomedical imaging^18–20^, segmentation^21^, prediction^22^, denoising^23^ and other linear and nonlinear inverse problems^14,24–26^. In our previous work, we demonstrated that deep learning can reconstruct fine biological structures such as dendritic spines from scattered TFM images^27^. Similarly, in this work, we present a deep learning-based inverse reconstruction method for DEEP-TFM.

Our proposed method is termed DEEP^2^. DEEP^2^ consists of a learning-powered inverse model that can reconstruct a de-scattered image from only 32 DEEP measurements (instead of 256 in our original work, DEEP). The model architecture is inspired by the UNet but modified to include a self-attention mechanism on the expanding path and a terminating reconstruction block. Generating paired DEEP-TFM and ground-truth images to train the DEEP^2^ inverse model is impractical. So we trained DEEP^2^ using simulated data. To simulate training data, we first carefully modeled the forward imaging process of the DEEP microscope. As a part of the forward model, we also modeled the optical behavior of scattering tissue using Monte Carlo simulations. We then used the forward model to simulate DEEP images from PSTPM-like image stacks (synthetically generated or experimentally acquired). The DEEP^2^, trained on the simulated data, was finally tested on the experimentally acquired DEEP images of fluorescent beads and mouse cortical vasculature. Our results suggest that the proposed method can reconstruct deep tissue images up to 4 scattering lengths (at the emission wavelengths)—accounting for ~200 μm—deep in mouse cortical vasculature, using only tens of patterned excitations instead of hundreds.

## Results

### Deep learning powered DEEP microscopy

**Fig. 1:**
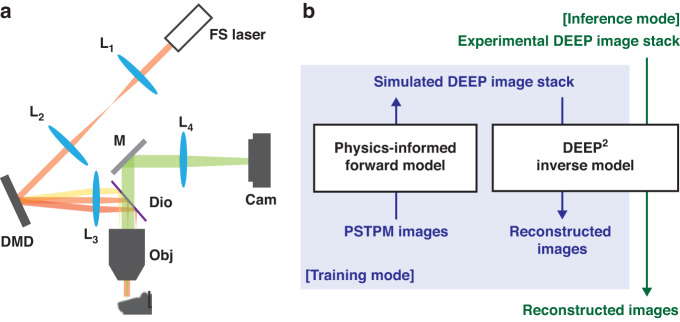
The proposed DEEP^2^ imaging method. **a** Optical schematic of the DEEP-TFM microscope: FS laser—amplified femtosecond laser; L1 and L2—optical relay; DMD—digital micro-mirror device; L3—excitation tube lens; Dio—dichroic mirror; Obj—objective lens; M—mirror; L4—emission tube lens; Cam—camera detector. **b** Schematic of the proposed physics-based forward model and the deep learning-based inverse model. In the training mode, point-scanning-two-photon microscopy (PSTPM) images are input to the physics-informed forward model to simulate their corresponding pixel-matched DEEP images. Then these paired simulated DEEP and PSTPM images are used to train the DEEP^2^ inverse model. In the inference mode, experimentally acquired DEEP images are input to the trained DEEP^2^ inverse model. The DEEP^2^ outputs reconstructions

In this work, we propose a deep-learning-based inverse model to reconstruct images from DEEP measurements. Figure 1a shows the optical schematic of the DEEP-TFM microscope. Amplified femtosecond laser pulses are temporally focused for depth-resolved multiphoton excitation. In the excitation path, a digital micro-mirror device (DMD) is placed at a conjugate image plane before the excitation tube lens. The DMD mirrors project a binary-patterned excitation onto the sample at the focal plane. The emission photons are collected by the objective lens and imaged onto a camera detector. During the experiment, multiple patterns generate multiple encoded image measurements. Our goal is to learn an inverse model to reconstruct de-scattered images using the knowledge about the excitation patterns and measured images (see Fig. 1b). To train such a model, we first generated simulated training data using a physics-based forward image model of the DEEP-TFM microscope and the scattering process. In the Methods section, we discuss the forward model, the electron multiplication CCD (EMCCD) detector model, and the scattering model in detail. The excitation patterns are parameters of the forward model. We first measured these patterns in a calibration experiment. Next, PSTPM-like ground-truth images were input to the forward model to simulate DEEP images (see Fig. 1b). This allowed us to generate paired DEEP and PSTPM data to train the inverse model. The inverse model was based on the seminal UNet architecture. We further modified the UNet by adding an attention mechanism to the expanding path. Details about the inverse model are presented in the Method section.

To test our method, we experimented on three datasets: (1) a mixture of fluorescent beads, (2) mouse pyramidal neurons with dendritic arbors, and (3) mouse cortical vasculature. We synthetically generated PSTPM-like beads images for training (see methods). Mouse pyramidal neurons and cortical vasculature were imaged from multiple animals using PSTPM to create the training datasets. We then performed physical DEEP experiments on a mixture of fluorescent beads and cortical vasculature of anesthetized mice (see Methods). We finally used our inverse model (trained on simulated data) to successfully de-scatter experimental DEEP measurements.

In the next two sections, we present numerical reconstruction results on unseen test datasets for each case; and de-scattering results for experimental DEEP measurements. We present all results in terms of “scattering lengths” or “scattering length-depths.” A scattering length is defined as the mean-free-path, i.e., the inverse of the scattering coefficient of the tissue at the emission wavelength. In experimental results, we assumed a scattering length of 50 μm^10,28^. Therefore 2, 4, and 6 scattering lengths, respectively, refer to 100, 200, and 300 μm.

### Numerical results on simulated test data

This section presents numerical results on simulated test datasets to validate our trained inverse model. All the experiments used only 32 patterned illuminations, which is nearly an order of magnitude less than the current state of the art^10^.

#### Reconstruction results on synthetic beads data

**Fig. 2:**
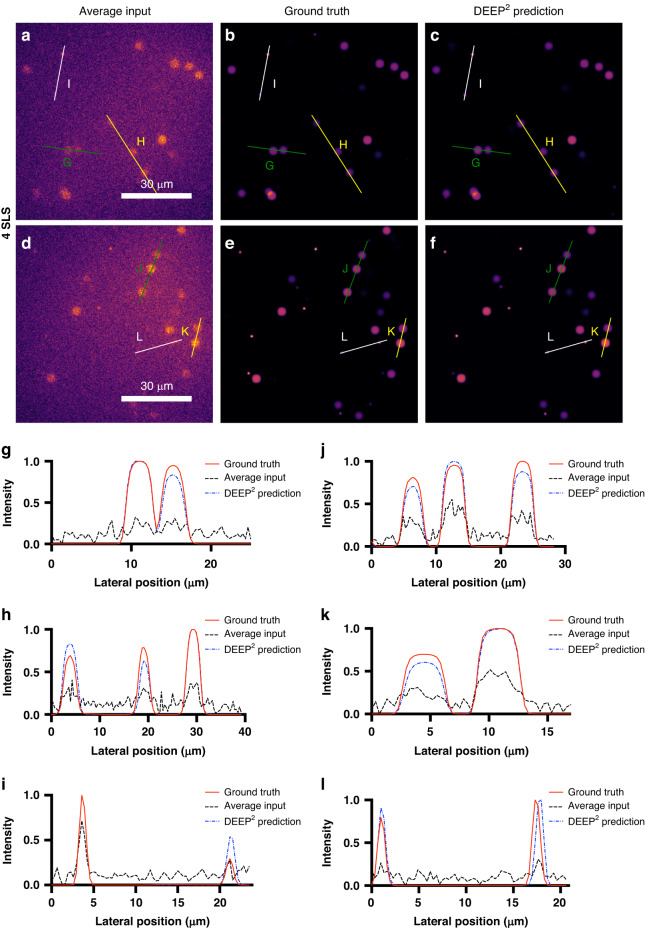
DEEP^2^ validation results on Synthetic Fluorescent Beads at 4 scattering lengths (SLS) below the surface. Synthetic beads-objects of the size of 1 and 4 μm, with the intensity of the 1 μm beads 5× higher than the 4 μm beads, were simulated. Their corresponding simulated DEEP-TFM image stacks were generated using the forward model. A subset of the data was used to train the DEEP^2^ inverse model, and the remaining unseen data were used to validate the model performance. **a**, **d** Two representative simulated DEEP-TFM image stacks (averaged over the 32 patterns). **b**, **e** Corresponding synthetic ground truths for the (**a**) and (**d**) instances. **c**, **f** DEEP^2^ reconstructions corresponding to (**a**) and (**d**) instances. The intensity along the lines G, H, and I shown in green, yellow and white on (**a**) is visualized in (**g**–**i**). Similarly, the intensity along the lines J, K, and L are shown in (**j**–**l**) plots

Synthetic 3D bead stacks were used as the input to the forward model to obtain the simulated DEEP-TFM-like instances at 4 scattering lengths. Then, the DEEP^2^-based inverse model was trained on the synthetic beads instances and was validated on similarly generated unseen synthetic beads instances. Figure 2 presents two validation instances, where the average input is shown in the first column, the ground truth in the second column, and the reconstruction of the DEEP^2^ in the third column. In addition, the intensities along the lateral directions (G, H, I, J, K and L in Fig. 2) were plotted for further clarification. The DEEP^2^ successfully reconstructed the small 1 μm beads (i.e., beads with 0.5 μm radius).

#### Mouse pyramidal neuron reconstructions

**Fig. 3:**
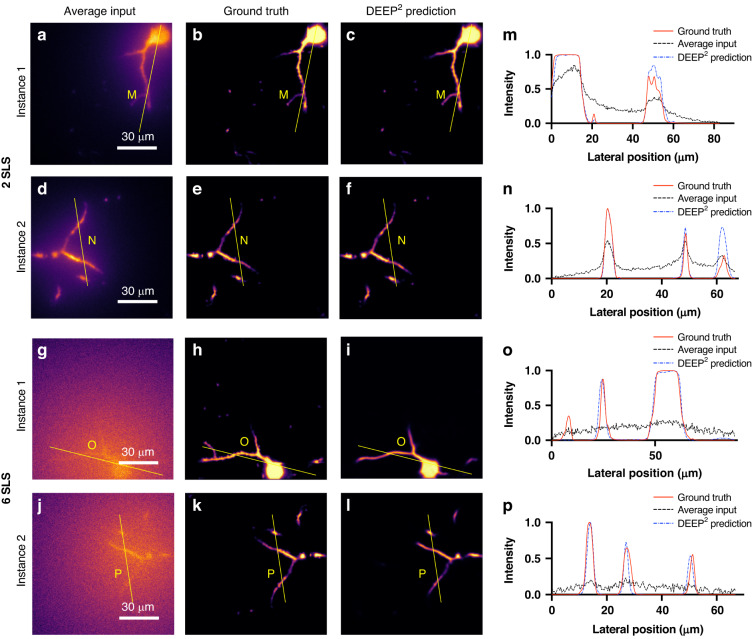
DEEP^2^ validation results on mouse pyramidal neurons with dendritic arbors at 2 and 6 scattering lengths (SLS) below the surface. PSTPM images of mouse pyramidal neurons were recorded. Their corresponding simulated DEEP-TFM image stacks were generated using the forward model. A subset of the data was used to train the DEEP^2^ inverse model, and the remaining unseen data were used to validate the model performance. **a**, **d**, **g**, **j** Four representative simulated DEEP-TFM image stacks (averaged over the 32 patterns) used for validation. **b**, **e**, **h**, **k** The corresponding PSTPM ground truths for the (**a**), (**d**), (**g**) and (**j**) instances. **c**, **f**, **i**, **l** DEEP^2^ reconstructions corresponding to (**a**), (**d**), (**g**) and (**j**) instances. The intensity along the yellow lines M, N, O, and P are visualized in (**m**–**p**) plots

Figure 3 shows the validation cohort results on the mouse pyramidal neurons with a dendritic arbor. We present two instances corresponding to 2 and 6 scattering length depths. The respective intensities along the lateral directions on each instance are shown in the last column. Figure 3a, d is two separate instances, each representing average of 32 patterned DEEP-TFM images generated using the forward model with the PSTPM mouse pyramidal neuron images at 2 scattering lengths. Figure 3b, e represents the PSTPM images, which were used as the ground truth for the corresponding Fig. 3a, d instances. Figure 3c, f is the reconstruction of DEEP^2^. The intensity along the yellow line on Fig. 3a–c and Fig. 3d–f are visualized in Fig. 3m, n graphs, respectively. Similarly, Fig. 3g, j shows an average of 32 patterned DEEP-TFM images synthesized using the forward model at six scattering lengths. Figure 3h, k shows corresponding ground truth, and Fig. 3i, l shows the reconstruction of DEEP^2^ at six scattering lengths. The intensity along the yellow line on Fig. 3g–i and Fig. 3j–l are represented on the Fig. 3o, p graphs, respectively.

#### Mouse cortical vasculature reconstructions

**Fig. 4:**
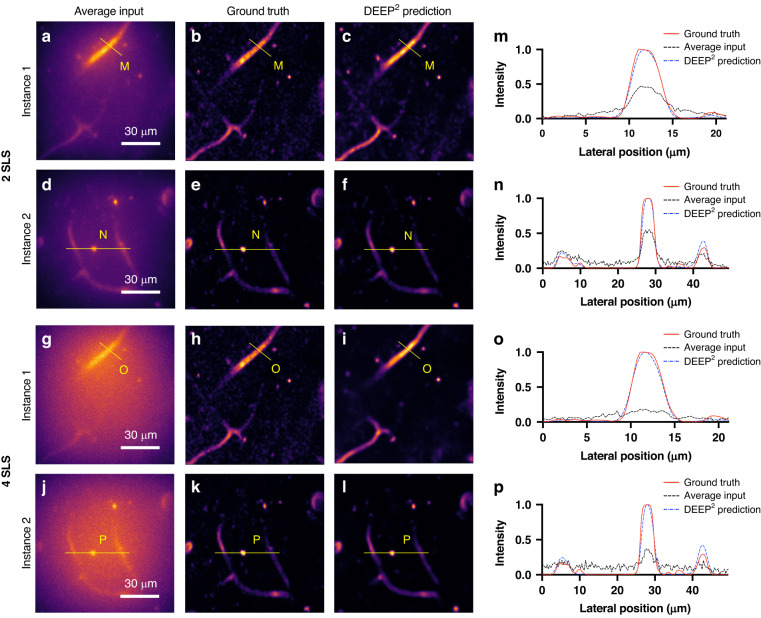
DEEP^2^ validation results on mouse cortical vasculature structures at 2 and 4 scattering lengths (SLS) below the surface. PSTPM images of mouse cortical vasculature were recorded. Their corresponding simulated DEEP-TFM image stacks were generated using the forward model. A subset of the data was used to train the DEEP^2^ inverse model, and the remaining unseen data were used to validate the model performance. **a**, **d**, **g**, **j** Four representative simulated DEEP-TFM image stacks (averaged over the 32 patterns) used for validation. **b**, **e**, **h**, **k** The corresponding PSTPM ground truths for the (**a**), (**d**), (**g**) and (**j**) instances. **c**, **f**, **i**, **l** DEEP^2^ reconstructions corresponding to (**a**), (**d**), (**g**) and (**j**) instances. The intensity along the yellow lines M, N, O, and P are visualized in (**m**–**p**) plots

Next, we trained DEEP^2^ to reconstruct mouse cortical vasculature images from numerical DEEP data. Figure 4 shows two validation instances at 2 and 4 scattering lengths below the surface. The intensity profiles along the highlighted lateral directions are presented in the last column. Figure 4a, d is the average of 32 patterned DEEP-TFM images, simulated using the forward model, for two separate mouse cortical vasculature instances. Figure 4b, e shows the corresponding ground truths. The DEEP^2^ reconstruction for the same two instances at 2 scattering lengths are presented in Fig. 4c, f. The intensity profiles along the marked yellow lines on Fig. 4a–c and Fig. 4d–f are indicated in Fig. 4m, n graphs, respectively. Similarly, for the same vasculature instances, the average over the 32 patterned DEEP-TFM instances synthesized using the forward model at 4 scattering lengths are shown in Fig. 4g, j. The corresponding ground truths are shown in Fig. 4h, k, and the DEEP^2^ reconstructions at 4 scattering lengths are shown in Fig. 4i, l. The intensity along the lateral directions marked in yellow on Fig. 4g–i and Fig. 4j–l are represented in Fig. 4o, p.

### Results on experimental data

In this section, we present experimental validation of DEEP^2^ by successfully de-scattering experimentally acquired DEEP measurements using the inverse model trained on simulated data. Experiments used only 32 patterned illuminations. Thus DEEP^2^ is nearly an order of magnitude faster than the current state of the art^10^.

#### DEEP^2^ imaging and reconstruction of a mixture of fluorescent beads

**Fig. 5:**
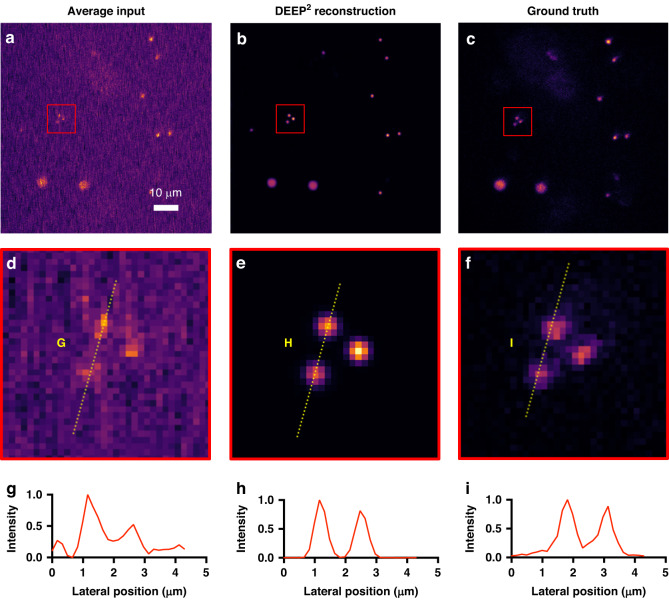
Experimental DEEP^2^ test results on fluorescent beads at 4 scattering lengths below the surface. A mixture of 1 and 4 μm beads was prepared and imaged through an intralipid layer using the DEEP-TFM microscope. The DEEP^2^ inverse model trained on simulated beads data was used in the reconstruction. **a** DEEP-TFM image stack averaged over the 32 patterns. **b** DEEP^2^ reconstruction corresponding to (**a**). **c** Ground truth image corresponding to (**a**), generated by imaging in the absence of intralipid. **d**–**f** The red colored box on (**a**–**c**) images are enlarged for close visualization. **g**–**i**. The normalized intensity along the yellow lines G, H, and I in (**d**–**f**) are visualized in (**g**–**i**) plots. DEEP^2^ reconstruction and ground truth are registered for visualization purposes

The DEEP^2^ inverse model trained on the artificial beads data at 4 scattering lengths was tested on experimentally acquired DEEP-TFM images. In accordance with the training data, similar-sized beads at a similar composition were imaged through a scattering intralipid layer (resulting in 4–5 scattering lengths) using the DEEP-TFM microscope with 32 patterned excitations. The images were then input to the trained inverse model to output the reconstructions. The results are shown in Fig. 5. Figure 5a shows the average of the 32 patterned DEEP-TFM images, and Fig. 5b shows the DEEP^2^ reconstruction. Figure 5c shows the ground truth acquired without the scattering intralipid layer. A region that contains 1 μm beads, marked as a red box on Fig. 5a, was closely observed to evaluate the performance. As shown in Fig. 5d, the average of 32 patterned DEEP-TFM images contained faint signs of those structures. But the proposed DEEP^2^ could reconstruct those fine beads, eliminating the background noise as shown in Fig. 5e. Finally, we also show the intensity cross-sections across the same beads, along the yellow lines drawn in Fig. 5d–f in Fig. 5g–i.

#### DEEP^2^ imaging and reconstruction of in vivo mouse cortical vasculature

**Fig. 6:**
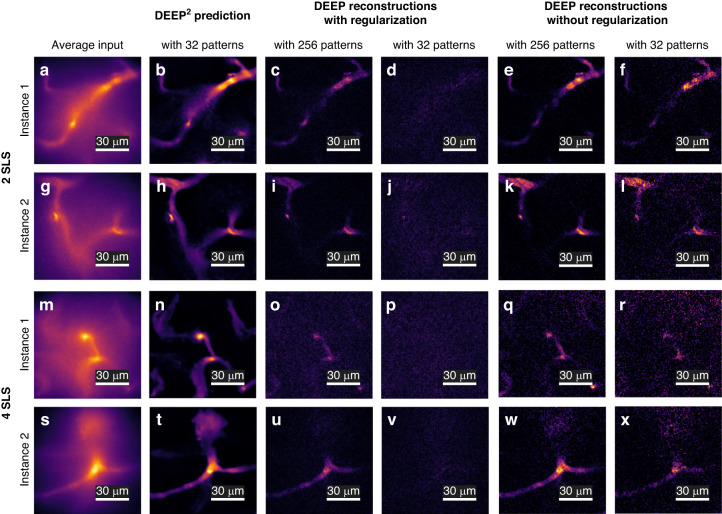
Experimental DEEP^2^ test results on mouse cortical vasculature structures at 2 and 4 scattering lengths (SLS) below the surface. The cortical vasculature of anesthetized mice with a cranial window was imaged using the DEEP-TFM microscope. The DEEP^2^ inverse model trained on simulated cortical vasculature data was used in the reconstruction. **a**, **g**, **m**, **s** Four representative instances of DEEP-TFM image stacks averaged over the 32 patterns. **b**, **h**, **n**, **t** DEEP^2^ reconstruction corresponding to (**a**), (**g**), (**m**), and (**s**)—with 32 patterned excitations. **c**, **i**, **o**, **u** Conventional DEEP reconstructions with regularization corresponding to (**a**), (**g**), (**m**), and (**s**)—with 256 patterned excitations. **d**, **j**, **p**, **v** Conventional DEEP reconstructions with regularization corresponding to (**a**), (**g**), (**m**), and (**s**)—with 32 patterned excitations. **e**, **k**, **q**, **w** Conventional DEEP reconstructions without regularization corresponding to (**a**), (**g**), (**m**) and (**s**)—with 256 patterned excitations. **f**, **l**, **r**, **x** Conventional DEEP reconstruction without regularization corresponding to (**a**), (**g**), (**m**) and (**s**)—with 32 patterns

Next, the DEEP^2^ model trained on simulated cortical vasculature data was tested on experimentally acquired DEEP-TFM images of mouse cortical vasculature (see Methods section for imaging details). Figure 6 shows several mouse cortical vascular instances at 2 and 4 scattering length depths. Figure 6a, g shows the average of the 32 patterned DEEP-TFM images for two separate FOVs at 2 scattering lengths. Figure 6b, h is the corresponding DEEP^2^ reconstructions with 32 DEEP-TFM images. Figure 6c, i are the DEEP-TFM reconstructions with 256 DEEP-TFM images using the original DEEP algorithm with a regularizer^10^. Figure 6d, j is the DEEP-TFM reconstruction with 32 DEEP-TFM images using the original DEEP inverse algorithm with the same regularizer^10^. We noticed that using a regularizer to remove noise may also remove dim image features (we also investigated this issue for DEEP^2^ as shown in Supplementary Fig. S14). Therefore, a similar DEEP reconstruction was also performed using a pseudo-inverse solver with no regularization (see corresponding results in Fig. 6e, f, k, l^10^).

Similarly, Fig. 6m, s shows the average of the 32 patterned DEEP-TFM images for two separate FOVs at 4 scattering lengths. Figure 6n, t is the DEEP^2^ reconstruction with 32 DEEP-TFM images corresponding to Fig. 6m, s, respectively. Figure 6o, u are the DEEP inverse algorithm reconstructions with regularization using 256 DEEP-TFM images at 4 scattering lengths. Figure 6p, v is the DEEP inverse algorithm reconstruction with regularization using 32 DEEP-TFM images at 4 scattering lengths. Figure 6q, r, w, x shows the same corresponding results without using the regularizer.

The DEEP^2^ reconstruction (with 32 DEEP-TFM images) showed additional details missing in the inverse algorithm reconstructions even with 256 DEEP-TFM images. This was more evident for DEEP reconstructions with a regularized inverse solver. However, the average of raw data (see Fig. 6a, g, m, s) suggests that these structures are real and not false artifacts generated by the network. The inverse algorithm with regularization failed to reconstruct with 32 DEEP-TFM images (see Fig. 6d, j, p, v), possibly due to a sub-optimal regularization loss term. But the inverse solver without a regularizer managed a noisy reconstruction for 2 scattering lengths.

## Discussion

In this paper, we developed a physics-informed-forward model and a deep-learning-based inverse model called DEEP^2^ to de-scatter DEEP-TFM images up to 4 scattering lengths below the surface. Our model uses only 32 DEEP-TFM raw images in the reconstruction, while the original DEEP used 256 such images. Therefore DEEP^2^ is almost an order of magnitude faster than DEEP. We validated our approach numerically and tested it experimentally to image in vitro samples through scattering media, as well as to image in vivo mouse cortical vasculature.

One major technical contribution of our work is the physics-based forward model that can simulate realistic DEEP-TFM images using PSTPM image data. This approach eliminates the need to collect paired PSTPM and DEEP data—which is nearly impossible for in vivo experiments—to train the neural network inverse model. The inverse model, trained on simulated data, was first validated on unseen simulated data (validation experiments). The same model was then tested on new experimental data (test experiments). In the validation experiments, the model worked up to six scattering lengths on multiple datasets (see Supplementary Figs. S2–S4). In in vivo test experiments, however, the model worked only up to 4 scattering lengths; the model failed at six scattering lengths (see Supplementary Fig. S6). This suggests a model mismatch between the forward model and the experimental system at higher scattering lengths. Therefore, an interesting future direction is to investigate reasons for the model mismatch in controlled test experiments using in vitro samples.

We tested the sensitivity of the inverse model to the changes in the scattering length. The models each trained on 2, 4, and 6 scattering lengths were tested on all 3 scattering lengths (see Supplementary Fig. S7). The models were indeed sensitive to the training conditions. We also further experimented with the model trained on 2 scattering lengths on validation data from a range of 1.6–2.4 scattering lengths. We observed a gradual drop in performance on off-training-data conditions (see Supplementary Fig. S10). To overcome this issue, we then trained a single model with a mix of scattering lengths (see results in Supplementary Fig. S7d, e). This model generalized to all scattering lengths in the training data mix. This observation suggests that a single model may be trained to generalize to a range of scattering lengths by mixing these conditions in the simulated training data. We also tested its sensitivity to off-training data conditions at 1.6–2.4 scattering lengths. The performance loss was lower than the model trained only on 2 scattering lengths training data (compare the red plot to the black—see Supplementary Fig. S10). The model trained on a mix also seemed to perform better at higher scattering lengths (see the last column in Supplementary Fig. S7e). Furthermore, we performed an ablation study to investigate the importance of different parts of the forward model (see results in see Supplementary Fig. S11). We observed that the EMCCD noise model and scattering model were critical parts of the simulated data generation process.

We also investigated the minimum number of patterns needed for reconstruction by training an ensemble of models that reconstruct from 1, 2, 4, 8, 16, and 32 patterns at 2, 4, and 6 scattering lengths. As expected, the minimum number of patterns needed for satisfactory performances depended on the scattering length. As shown in Supplementary Fig. S9e for the 2-scattering-length mouse cortical vasculature data, a stable and satisfactory performance was achieved even with four patterns. However, 6-scattering-length mouse cortical vasculature data required a minimum of 32 patterns to perform satisfactorily. We believe that a higher number of patterns could give better performance for deeper scattering lengths. But there is a tradeoff between performance and computational requirements, and we were not able to successfully experiment with a higher number of patterns. As a conclusion, we chose 32 as the number of patterns considering the computational limitations and the model’s performance with the different number of patterns. We also extensively compared the original DEEP^10^ under the same conditions (see Supplementary Figs. S12 and S13). In numerical validations, DEEP could reconstruct marginally satisfactorily at 2 scattering lengths with 32 patterns (see structural similarity index (SSIM), peak signal-to-noise ratio (PSNR), and qualitative results in Supplementary Fig. S12b, d). But at 4 and 6 scattering lengths, DEEP reconstructions were not satisfactory (see Supplementary Fig. S12). We observed similar trends in the DEEP reconstruction on experimental cortical vasculature data (Supplementary Fig. S13). This benchmarking experiment clearly demonstrates the power of DEEP^2^ compared to DEEP.

We also tested the effect of architecture and cost function of the inverse model with respect to the forward model mismatch. To this end, we tested five variations of the inverse model on three datasets. The validation experiments did not show significant differences in qualitative and quantitative performance among the five variations (see Supplementary Figs. S1–S4). The test experiments on real data, however, showed that the combination of KL divergence loss with the channel and spatial attention mechanism (scSE-UNet) helps with the forward model mismatch (see Supplementary Figs. S5 and S6). Further numerical experiments and interpretation are needed to definitively interpret the reasons for this behavior with different forward model variations. Our current DEEP^2^ approach requires training a separate inverse model for every forward model variation. Therefore, testing a wide range of forward model variations is not realistic. A future direction to overcome this limitation is to develop forward-model-agnostic inverse approaches using deep generative priors. Such an approach will not require re-training a separate inverse model for each forward model variation; hence, one can run many numerical experiments to investigate the model mismatch problem. This will also help investigate other behaviors, such as the effect of the number of DEEP-TFM raw images in the reconstruction and the effect of the excitation pattern selection. In terms of inverse model selection, another interesting direction is exploring efficient architectures that can be trained for more patterns than 32.

For experimental results, some DEEP^2^ reconstructions looked visually better than the DEEP reconstructions using even full 256 patterns. We think this is mostly because the DEEP^2^ inverse network acting as a denoizer (in addition to a de-scatterer). For DEEP, we attempted some denoizing using regularized inverse solvers with limited success. Moreover, DEEP^2^ (as well as DEEP) demonstrated enhanced depth sectioning that could make the reconstructed results look better than the pseudo ground truth results (for example, in the case of the beads). This is because DEEP^2^ (as well as DEEP) uses structured (i.e., patterned) illumination that may lead to better depth sectioning compared to widefield illumination used to generate the pseudo ground truth. Nevertheless, further controlled experiments are needed to definitively rule out the possibility of false reconstructions due to model overfitting. Finally, while we have improved the speed of DEEP^2^ by almost an order of magnitude compared to DEEP, both DEEP^2^ and DEEP still require further optimization in instrument design to outperform traditional point-scanning and line-scanning systems. DEEP^2^ uses an EMCCD detector that is orders of magnitude slower than photomultiplier tubes (PMTs) used in point-scanning systems. To improve the practical speed in DEEP^2^, the next-generation instrument needs to be built with fast sensors like multi-anode PMTs or avalanche photodiode arrays. Nevertheless, these instrumentation advances are orthogonal to the algorithmic advanced presented in this work. Hence, DEEP^2^ can improve the speed of any potential DEEP system with advanced optical designs.

In conclusion, in this work, we demonstrate DEEP^2^, a physics-informed forward model, and a deep-learning-based inverse model for DEEP-TFM. DEEP^2^ is an order of magnitude faster than the original DEEP^10^. We validated our approach on unseen numerical data, as well as experimentally acquired test data. We demonstrate that DEEP^2^ can de-scatter in vivo cortical vasculature images up to 4 scattering lengths below the surface. Our work set the foundation to develop data-driven inverse solvers for DEEP-TFM using deep learning, and hence an important milestone for in vivo widefield multiphoton microscopy.

## Materials and methods

### Forward model

#### Model architecture

**Fig. 7:**
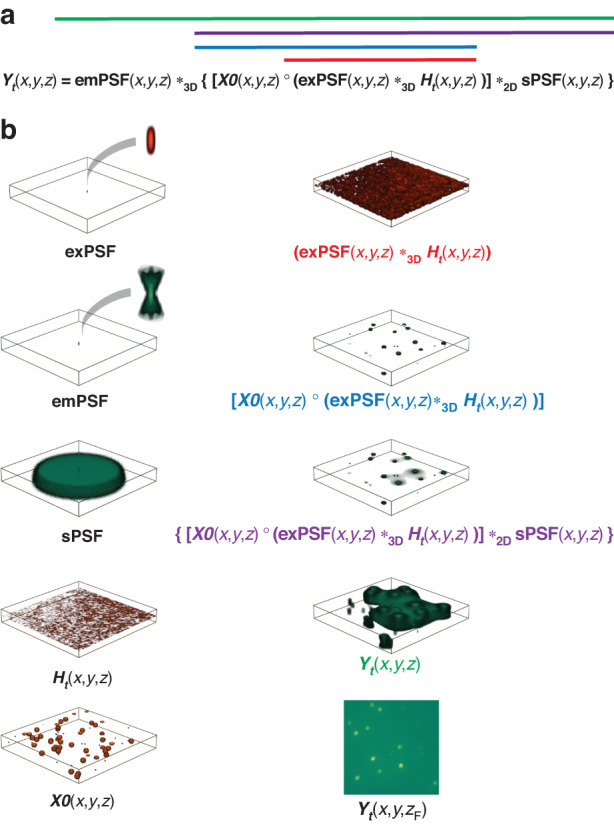
Visualization of the physics-informed forward model. **a** The forward model equation. **b** A representative simulation of the forward model for a 3D beads specimen. Note that the different sections of the equation in (**a**) are shown in color-coded bars on the equation in (**b**)

To be used as inputs to our deep learning model for training, we generate DEEP-TFM image stacks synthetically from the PSTPM image stacks using a forward imaging model (see Fig. 7). Let *exPSF*(*x*, *y*, *z*) be the 3D excitation point spread function (PSF) and *H*_*t*_(*x*, *y*) be the pattern on the DMD placed at an image plane at the excitation side. Now, *exPSF*(*x*, *y*)*_3*D*_*H*_*t*_ is the 3D patterned excitation on the sample. Here *_3*D*_ is the 3D convolution operation. The result is multiplied element-wise with the object, *X*0(*x*, *y*, *z*) to get the excited object cube. Then the resulting 3D excited object is convolved plane-by-plane with the stack of 2D scattering PSFs at each depth (*sPSF*(*x*, *y*, *z*)). This operation is represented by *_2*D*_. Each *sPSF* at a certain depth, represents the effective scattered light distribution from a point source at that depth. We introduce and discuss the sPSFs model in the next section in detail. The resulting 3D cube is then convolved in 3D with the emission PSF (*emPSF*(*x*, *y*, *z*)). These operations constitute the following equation:

1 $${Y}_{t}(x,y,z)=\{\{(exPSF(x,y,z)\ {* }_{3D}\ {H}_{t}(x,y))\ \circ \ X0(x,y,z)\}\ {* }_{2D}\ sPSF(x,y,z)\}\ {* }_{3D}\ emPSF(x,y,z)$$

Finally, we get the image on the detector, by selecting the *z*-plane that corresponds to the focal plane (*z*_*focal*_) from the last output cube as shown in Fig. 7. Then the noise is added to the resulting image using the following noise model discussed in the “EMCCD noise model” section.

2 $${\hat{Y}}_{t}(x,y)={f}_{EM}( \sim Poiss({Y}_{t}(x,y,{z}_{focal}))+ \sim Poiss({\bar{Y}}_{Dark}))+ \sim Norm(0,{\sigma }_{Read})$$

**Fig. 8:**
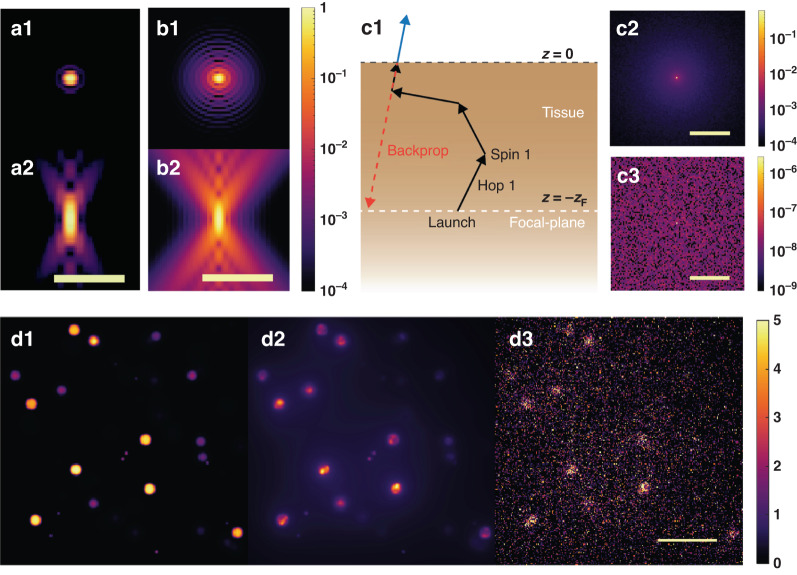
A simulation from the physics-informed forward model. **a1–2** The *xy* and *xz* views of the excitation PSF. **b1–2** The *xy* and *xz* views of the emission PSF. **c1** Illustration of the light scattering process in a scattering tissue used to model the scattering point spread function (sPSF). **c2** The scattering point spread function at a two-scattering-length depth. **c3** The scattering point spread function at a seven-scattering-length depth. **d1** A synthetic beads object (the maximum intensity projection over the *z*-axis). **d2** The simulated DEEP-TFM image of the object in (**d1**) 7 scattering lengths below the surface (before detection on the camera). Note that the maximum photon count in the image is close to 5 photons. **d3** The simulated DEEP-TFM image in (**d2**) detected on the simulated EMCCD camera. The scale bars in (**a2**), (**b2**), (**c2**), and (**c3**) are 5 μm. The scale bar in (**d3**) is 20 μm

Here, ~*Poiss*(*μ*) denotes the observations drawn from a Poisson distribution of mean *μ*; ~*Norm*(*μ*, *σ*) denotes the observations drawn from a Normal distribution of mean *μ* and standard deviation *σ*; ${\bar{Y}}_{Dark}$ is the expected value of dark current of the camera and *f*_*EM*_(.) models the electron multiplying (EM) process of the EMCCD camera. Figure 8 shows the cross-sections of the simulated PSFs and representative simulated DEEP-TFM images.

For the beads dataset, fully simulated objects were used as *X*0s. For mouse pyramidal neuron and mouse cortical vasculature datasets, experimentally acquired PSTPM images were used as *X*0s after pre-processing them to match the voxel size of the DEEP-TFM experiment. During training, the PSTPM images were simulated and used as the output (or the ground truth) of the network. The object *X*0 was convolved with the excitation PSF to generate the PSTPM image.

#### Scattering point spread function (sPSF)

In this section, we discuss the mathematical model of the scattering PSF. We define the scattering PSF as the effective photon distribution of a certain *z*-plane below the tissue surface due to a point source placed at that *z*-plane. Using Monte Carlo simulations of light transport through scattering tissue from a point source, we model how the downstream optics see the light distribution from the plane where the point source is located. Figure 8c1 illustrates how the light scattering process is modeled using Monte Carlo methods. The surface is treated as the *z* = 0 axial plane. A fluorescent light source is placed at *z* = −*z*_0_ plane. The lateral location of this point source is considered as *x* = *y* = 0. A photon is then launched from the source, placed at (0, 0, −*z*_0_), in the random direction (*u*_*x*_, *u*_*y*_, *u*_*z*_). This photon is propagated for s distance (call this a hop). Then a scattering event occurs, and the direction of the photon is changed by a deflection angle, *θ*, and an azimuth angle, *ψ* (call this a spin). Then the photon is hopped and spun repeatedly until it reaches the surface. Once it reaches the surface, the photon’s final direction is recorded. If the direction is within the range captured by the numerical aperture of the objective, the photon is then backpropagated until it reaches the original launch plane (i.e., *z* = −*z*_0_), and its location on the launch plane is recorded. When the imaging system is focused on this launch plane, the photon appears to have originated from this location. This process is repeated a sufficiently large number of times to generate the sPSF at the launch plane.

In the simulation, we need to randomly generate the hop distance (*s*) and spin angles (*θ* and *ψ*) according to the scattering properties of the tissue. First, the probability distribution of *s* is related to the scattering coefficient of the tissue, *μ*_*s*_. By the definition of *μ*_*s*_^29^, the probability of transmission of a photon without encountering a scattering event after path-length *s* is given by,

3 $$P(S > s)={e}^{-{\mu }_{s}s}$$

Therefore, we can derive the cumulative distribution function (c.d.f.) of *s*, *F*(*s*), and probability density function (p.d.f.) of *s*, *f*(*s*) as follows:

4 $$F(s)=P(S\leq s)=1-{e}^{-{\mu }_{s}s}$$

5 $$f(s)=\frac{d(F(s))}{dt}={\mu }_{s}{e}^{-{\mu }_{s}s}$$

In Monte Carlo methods, *f*(*s*) is sampled using a computer-generated, uniformly distributed random number *rnd*_1_ such that,

6 $$rn{d}_{1}=F(s)$$

Therefore, we can derive *s* as a function of *rnd*_1_ as follows:

7 $$s=-{\rm{ln}}(1-rn{d}_{1})/{\mu }_{s}$$

Second, the deflection angle, *θ*, is related to the anisotropy, *g*, of the tissue according to the Henyey–Greenstein scattering function (HG function) that mimics the angular dependence of light scattering by small particles^29^. According to the HG function, the p.d.f. of cos(*θ*) can be written as follows:

8 $$f({\rm{cos}}(\theta ))=\frac{1}{2}\frac{(1-{g}^{2})}{{(1+{g}^{2}-2g\,{\rm{cos}}(\theta ))}^{(3/2)}}$$

From the same principles of sampling *f*(cos(*θ*)) using a second random number *rnd*_2_ it can be shown that,

9 $${\rm{cos}}(\theta )=\frac{1}{2g}\left(1+{g}^{2}-{\left(\frac{1-{g}^{2}}{1-g+2grn{d}_{2}}\right)}^{2}\right)$$

Last, the azimuth angle, *ψ*, can be picked at random, i.e., *ψ* = *rnd*_3_, where *rnd*_3_ is a third computer-generated normally distributed random number. For more details, we refer interested readers to Jacques and Wang^29^.

#### EMCCD noise model

Next, we model the noise added to the image, as shown in Eq. (2). We use an EMCCD camera for detection. Let *Y*_*Shot*_ be the number of electrons $\bar{e}$ from the signal detected before the EM process. Note that the expected value of *Y*_*Shot*_ (i.e., ${\bar{Y}}_{Shot}$) is equal to *Y*_*t*_(*x*, *y*, *z*_*focal*_) in Eq. (2). Let *Y*_*Dark*_ be the number of electrons generated by the dark current of the camera. Dark current is usually listed in the camera specifications in “$\bar{e}/pixel/s$” and can be used to calculate the expected value of *Y*_*Dark*_ (i.e., ${\bar{Y}}_{Dark}$) when the exposure time is known. Both *Y*_*Shot*_ and *Y*_*Dark*_ are Poisson distributed. Let *N*_*Read*_ be the read noise of the camera. The standard deviation of the read noise (*σ*_*Read*_) is listed in the camera specifications in $\bar{e}$. Read noise can be described by a normal distribution with zero mean and (*σ*_*Read*_) standard deviation. Let us denote the EM process by a statistical function *f*_*EM*_(.). Then we can write a formulation for the output signal, *Y*_*Out*_ (equals to ${\hat{Y}}_{t}(x,y)$ in Eq. (2)), in $\bar{e}$ as follows:

10 $${Y}_{Out}={f}_{EM}({Y}_{Shot}+{Y}_{Dark})+{N}_{Read}$$

We can then write the mean and variation equations for the signals from Eq. (10) as follows:

11 $${\bar{Y}}_{Out}={g}_{EM}({\bar{Y}}_{Shot}+{\bar{Y}}_{Dark})$$

12 $${\sigma }_{Out}^{2}={g}_{EM}^{2}{F}^{2}({\sigma }_{Shot}^{2}+{\sigma }_{Dark}^{2})+{\sigma }_{Read}^{2}$$

Here $\bar{Y}$ denotes the mean of the random variable *Y*. *g*_*EM*_ is the EM gain of the camera, and it is the average gain added by the EM process. The range of *g*_*EM*_ is listed in camera specs and the exact value is set during imaging. *F* quantifies the noise added by the EM process, *f*_*EM*_(.), and is discussed in the next subsection.

#### Electron multiplication process, *f*_*EM*_(.)

The EM process in practice is noisy and *g*_*EM*_ is only the average value of the EM gain. This noise is quantified by the excess noise factor, *F*, defined in Eq. (12). We can derive an expression for *F*^2^ from Eq. (12) as follows:

13 $${F}^{2}=\frac{{\sigma }_{Out}^{2}-{\sigma }_{Read}^{2}}{{g}_{EM}^{2}\left({\sigma }_{Shot}^{2}+{\sigma }_{Dark}^{2}\right)}$$

Here $({\sigma }_{Shot}^{2}+{\sigma }_{Dark}^{2})$ is the variation of the signal that goes into the EM process, and $({\sigma }_{Out}^{2}-{\sigma }_{Read}^{2})$ is the variation of the signal that comes out of the EM process (if F is 1 there is no noise added to by the EM process). Robins and Hadwen^30^ derived an expression for *F* as follows:

14 $${F}^{2}=\frac{1}{{g}_{EM}}\left(\frac{2{g}_{EM}+\alpha -1}{\alpha +1}\right)=2({g}_{EM}-1){g}_{EM}^{(-(N+1)/N)}+\frac{1}{{g}_{EM}}$$

Here, *N* is the number of EM gain stages. EM process is treated as a Bernoulli process at each EM stage, and *α* is the probability of an electron multiplication event happening, i.e., the probability of a success event. The value of a is usually small and is in the order of 1–2%^31^. Considering all input electrons to a stage, the gain for each EM stage can be described by a Binomial distribution with a probability mass function,

15 $$pmf({x}_{Ad};{x}_{in},\alpha )=Pr({X}_{Ad}={x}_{Ad})=\left(\begin{array}{c}{x}_{in}\\ {x}_{Ad}\end{array}\right){\alpha }^{{x}_{Ad}}{(1-\alpha )}^{({x}_{in}-{x}_{Ad})}$$

Here *x*_*in*_ is the number of input electrons to the gain stage, and *X*_*Ad*_ is the number of added electrons through the EM gain stage. The output electrons from the gain, therefore, equal to *X*_*Ad*_ + *x*_*in*_. *N* such gains stages are cascaded to get the final EM output signal *f*_*EM*_(.).

However, simulating a cascade of *pmf*(.) functions of the form Eq. (15) to add noise to every pixel in every simulated image is prohibitively slow. Therefore, for each potential input value to *f*_*EM*_(.), we generated a distribution of *f*_*EM*_(.) output values. Note that this is doable as the inputs to *f*_*EM*_(.) are integers that come from Poisson distributed variables (i.e., *Y*_*Shot*_ + *Y*_*Dark*_). Then during the simulations, we randomly sampled the output distribution corresponding to the input value to get the output of *f*_*EM*_(.) in Eq. (10).

### Inverse model

#### Network architecture

**Fig. 9:**
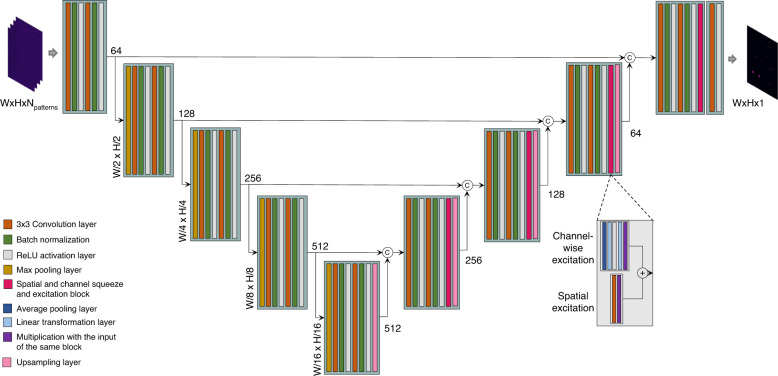
The deep-learning-based inverse model architecture. UNet with concurrent channel and spatial attention mechanism employed in the proposed DEEP^2^ inverse model

In this work, we use a deep learning network, called DEEP^2^, as the inverse model. Our proposed DEEP^2^ is inspired by the UNet^32^ architecture with some modifications, as illustrated in Fig. 9. DEEP^2^ consists of 9 convolution blocks, each block containing two convolutional layers followed by batch normalization and a ReLU activation. In the encoder block, the output of each block is sent through a max pooling layer for downsampling. An additional block, which we call scSE, is integrated into the decoder blocks. The importance of this block is discussed in the next section. The feature maps are upsampled in the decoder path before feeding into the next decoder block. The skip connections between the downsample and upsample parts enable the model to learn the fine-grained details in the biological structures more efficiently. We exploit a separate block at the end, which consists of a convolutional layer and a ReLU activation layer, as the final reconstruction step.

#### UNet with spatial and channel squeeze and excitation

In any deep learning model, the main purpose of the convolution layer is to learn to capture all local spatial information within each channel and to create feature maps jointly encoding the spatial and channel dependencies. Improving the joint encoding of spatial and channel information through convolution requires much effort. Thus, the Concurrent Spatial and Channel “Squeeze and Excitation” (scSE) block is introduced^33^. This improves the feature map by suppressing the weak features and enhancing the important features. Despite this, recalibrating non-significant features towards zero with scSE may increase the sparsity in deeper layers as well as reduce the parameter learning^34^. We denote the performance enhancement of the UNet with scSE over the vanilla UNet in our experiments.

#### Loss function

Our learning algorithms focus on solving the optimization problem given by structural risk minimization, $\mathop{\min }\nolimits_{f}\frac{1}{N}\mathop{\sum }\nolimits_{i = 1}^{N}{L}_{\theta }({I}_{f}{\hat{Y}}_{t},X0)$, where ${\hat{Y}}_{t}$ is the set of DEEP-TFM images given to the Inverse model *I*_*f*_ and the *X*0 is the corresponding ground truth (unscattered) image. In the past few years, the attention of the machine learning community has been drawn to the selection of the loss function, which provides a significant theoretical and practical value to the optimization task. Therefore, we have exploited four loss functions with the above-described architecture as the inverse model to obtain the optimal learning algorithm. The least squared error given by Eq. (16) is the most frequently used loss function in regression tasks. Let us assume the output of the inverse model is $\hat{X0}$,

16 $$Los{s}_{MSE}=\frac{1}{n}\mathop{\sum }\limits_{t=1}^{n}{\left(X0-\hat{X0}\right)}^{2}$$

This loss function can converge fast as the gradient of the loss decreases when the loss approach 0. However, a drawback of this loss function is the high sensitivity for outliers. Root Mean Squared Logarithmic Error (RMSLE) is more robust to the outliers as it nullifies the effect by considering the $\log$ value.

17 $$Los{s}_{RMSLE}=\sqrt{\frac{1}{n}\mathop{\sum }\limits_{t=1}^{n}{\left(\log \left(X0\right)-\log \left(\hat{X0}\right)\right)}^{2}}$$

Therefore, the above loss function is also utilized in our experiments. Next, we also explored the Smooth L1 loss, which is also more robust for the outliers compared to the mean squared error (MSE).

18 $$Los{s}_{SmoothL1}=\left\{\begin{array}{ll}\frac{1}{n}\mathop{\sum }\nolimits_{t = 1}^{n}\frac{1}{2}{\left(X0-\hat{X0}\right)}^{2}&\,{{\mbox{if}}}\,\,\frac{1}{n}\mathop{\sum }\nolimits_{t = 1}^{n}\left\vert X0-\hat{X0}\right\vert < 1\\ \frac{1}{n}\mathop{\sum }\nolimits_{t = 1}^{n}\left\vert X0-\hat{X0}\right\vert -\frac{1}{2}&\,{{\mbox{otherwise}}}\,\end{array}\right.$$

Smooth L1 loss conditionally chooses either the mean squared loss or the absolute loss, while combining the advantages of both loss functions. Finally, we also explored the usability of KL divergence as a loss function, which can handle distributions more efficiently despite the normality of the approximation error.

19 $$Los{s}_{KLDivergence}=\frac{1}{n}\mathop{\sum }\limits_{t=1}^{n}X0\cdot \log \left(\frac{X0}{\hat{X0}}\right)$$

### Training and evaluating the inverse model

The inverse model was trained using the Adam optimizer with learning rate (*lr*), *beta*1, *beta*2 and batch size set to, 10^−3^, 0.9, 0.999 and 10, respectively, for 100 epochs. All the experiments are conducted with the PyTorch^35^ learning framework. All the experiments are done with the Titan RTX available on the FASRC Cannon cluster at Harvard University.

#### Evaluation metrics

We mainly used three metrics to evaluate the performance of our model on the validation data (see supplementary): (1) SSIM^36^, (2) MSE, (3) PSNR. The quantitative evaluation of the proposed model on the validation dataset is presented in Table 1.

20 $$SSIM=\frac{(2{\mu }_{x}{\mu }_{y}+{C}_{1})(2{\sigma }_{xy}+{C}_{2})}{({\mu }_{x}^{2}+{\mu }_{y}^{2}+{C}_{1})({\sigma }_{x}^{2}+{\sigma }_{y}^{2}+{C}_{2})}$$

21 $$MSE=\frac{1}{n}\Sigma {({x}_{i}-{y}_{i})}^{2}$$

22 $$PSNR=10{\log }_{10}\left(\frac{{R}^{2}}{MSE}\right)$$

where ${\mu }_{x},{\mu }_{y},{\sigma }_{x}^{2},{\sigma }_{y}^{2},{\sigma }_{xy}$ are mean, standard deviation, and covariance of ground truth image *x* and output of the inverse model *y*. $C1={({k}_{1}\times L)}^{2}$ and $C2={({k}_{2}\times L)}^{2}$ are regularization parameters with *L* = 1.0, *k*_1_ = 0.01 and *k*_2_ = 0.03. *n* denotes the number of pixels in the image, and *R* denotes the maximum intensity of the pixels in input images.

**Table 1:** Quantitative comparison (MSE/PSNR/SSIM) of the proposed model with KL divergence loss

Dataset	SLS	Evaluation metrics		
		MSE (10^−4^)	PSNR	SSIM (10^2^)
Mouse cortical vasculature	2 SLS	4.33 ± 2.46	34.11 ± 1.87	92.45 ± 1.97
	4 SLS	6.77 ± 3.01	32.03 ± 1.62	88.79 ± 2.08
	6 SLS	29.07 ± 12.71	25.72 ± 1.72	80.76 ± 3.86
Mouse pyramidal neuron	2 SLS	2.76 ± 1.49	36.18 ± 2.28	97.10 ± 1.29
	6 SLS	15.46 ± 9.51	28.99 ± 2.89	85.81 ± 4.74
Synthetic beads	4 SLS	1.33 ± 0.58	39.16 ± 1.82	78.75 ± 12.95

### Training and validation datasets

#### Artificial fluorescent beads mixture dataset

We experimentally acquired fluorescent beads images using our DEEP-TFM system to test DEEP^2^. To train DEEP^2^ inverse model under similar conditions, we simulated a synthetic dataset. Artificial beads data, in the size of the experimentally acquired DEEP-TFM instance, were generated to use as the input to the forward model. Here 3D volumes with beads in radius 0.5 μm (1.5 pixels) and 2 μm (6 pixels) were generated along with randomly selected intensities from a normal distribution with mean 1 and standard deviation 0.1. The 0.5 μm radius beads intensities were enhanced by 5 times compared to the 2 μm radius beads. These artificially generated 3D volumes with beads were given as the input to the forward model to generate the simulated DEEP-TFM instances. Since the experimental beads data acquired using DEEP-TFM was obtained at 4 scattering lengths, the same depth was used for the synthetic bead dataset creation. Thus, for 4 scattering lengths, 3455 instances were obtained, which we split into a 4:1 ratio for training and validation. In addition, 128 artificial beads instances were generated for independent testing. Each instance consists of 32 patterned DEEP-TFM-like images of size 256 pixels by 256 pixels.

#### Mouse pyramidal neuron dataset

Experimentally obtained images of pyramidal neurons with the dendritic arbor, using a PSTPM, were also used for simulated DEEP-TFM image generation using the forward model. Each neuronal image volume was 607 pixels × 607 pixels × 263 pixels with a spatial resolution of 0.25 μm. We used 11 such neuronal image volumes to create simulated DEEP-TFM neuronal instances. To enhance the intensity of the fine structures such as dendritic spines, the intensity was thresholded at 20, by assigning 20 for all the pixels with intensity over 20. The successive images along the *z*-plane of the image volume contained similar structures. Thus, to avoid repeating similar training instances, the sub-image volumes that match the third dimension of the exPSF, were extracted from the neuronal volume, to use as the input to the forward model, with 5 image planes apart from each other. Furthermore, to avoid the image volumes with no significant structures, the sub-image volumes with the mean exceeding the mean of the whole neuronal image volume were considered as the input of the forward model. The 3895 simulated DEEP-TFM neuronal instances were generated for 2 (~100 um) and 6 (~300 um) scattering lengths. Each instance consists of 326 pixels by 326 pixels ground truth (${\hat{X}}_{0}(x,y)$) and 32 patterned images (*t* = 32) of size 326 pixels by 326 pixels (${\hat{Y}}_{t}(x,y)$) as the input of the inverse model.

#### Mouse cortical vasculature dataset

In addition to the experimentally acquired beads, we also imaged mouse cortical vasculature at increasing depths using the DEEP-TFM. Hence, to train the network, we used an equivalent vascular structural image volume acquired using a three-photon Fluorescent microscope^37^. The vascular image stack consists of 800 *z*-planes, each with an increment of 1.5 μm and each sized 512 pixels × 512 pixels with a spatial resolution of 0.6 μm corresponding to a field of view of ~300 μm.

#### Artificial vasculature dataset

In addition, artificial blood vessel volumes were employed^38,39^ to demonstrate the applicability of DEEP^2^ on vasculature structures furthermore. The synthetic volumes originally consisted of blood vessels with radii ranging from 1 to 7 pixels. The finest blood vessels with low intensities were removed by applying a threshold at 190. The blood vessel volumes were originally sized 325 pixels by 304 pixels by 600 pixels, and 20 such volumes were downloaded. Each volume was rescaled by factor 1.09, normalized by dividing by the maximum intensity of each volume and extracted volumes of 326 pixels by 326 pixels by 200 pixels volumes to redirect as the input to the Forward model. Similarly, we generated datasets at 2 and 6 scattering lengths for blood vessels.

### Mouse surgery and imaging

Experiments were carried out under protocols approved by MIT’s Animal Care and Use Committee and conformed to NIH guidelines. All data in this study were collected from adult (>8 weeks old) mice of either sex. The wild-type mice were acquired from the Jackson laboratory (#000664). Mice were initially anesthetized with 4% isoflurane in oxygen and maintained on 1.5–2% isoflurane throughout the surgery. Buprenorphine (1 mg kg^−1^, subcutaneous) and/or meloxicam (1 mg kg^−1^, subcutaneous) was administered preoperatively and every 24 h for 3 days to reduce inflammation. Ophthalmic ointment was used to protect the animal’s eyes during the surgery. Body temperature was maintained at 37.5 °C with a heating pad. The scalp overlying the dorsal skull was sanitized and removed. The periosteum was removed with a scalpel, and a craniotomy (5 mm) was made over the primary visual cortex (V1, 4.2 mm posterior, 3.0 mm lateral to Bregma) on either the left or right hemisphere, leaving the dura intact. Last, a custom-designed stainless steel head plate (eMachineShop.com) was affixed to the skull using dental acrylic. Experiments were performed at least 5 days after head plate implantation to allow animals to recover. For labeling blood vessels with a fluorescent dye, a rhodamine + dextran dye (70 kDa; D1841, Thermo Fisher Scientific) mixed with saline solution at 5% (wv^−1^) concentration was applied retroorbitally with 100 μl volume^40^. During the retroorbital injection, the animal was anesthetized with 2% isoflurane in oxygen. During both 2-photon and 3-photon imaging, the animal was anesthetized with ketamine + xylazine mixture with 0.1 ml volume, and this mixture was applied as needed after checking the reflexes. The imaging sessions lasted for a maximum of 2 h.

### Supplementary information

Supplementary Information for DEEP-squared: Deep Learning Powered De-scattering with Excitation Patterning

## Data Availability

The dataset and excitation patterns used in the forward model of this study are available on https://zenodo.org/record/8161051. The datasets simulated by the forward model are available from the corresponding author upon request.

## Code availability

The codes used in this study are available through a public GitHub repository: https://github.com/wadduwagelab/DEEP-squared.
