# Supplementary material for: DEEP-squared: deep learning powered De-scattering with Excitation Patterning
Source: Light Sci Appl. 2023 Sep 13;12:228. doi: 10.1038/s41377-023-01248-6 (PMC10499829; doi:10.1038/s41377-023-01248-6)
Supplement: Supplementary file 1 — Supplementary Information for DEEP-squared: Deep Learning Powered De-scattering with Excitation Patterning [file 41377_2023_1248_MOESM1_ESM.pdf]

Supplementary Information for DEEP-squared: Deep Learning Powered De-scattering with Excitation Patterning

Navodini Wijethilake[1,2,†], Mithunjha Anandakumar[1,†], Cheng Zheng[3,4], Peter T. C. So[3,4,5], Murat Yildirim[4,6,7], and Dushan N. Wadduwage[1,*]

[1]Center for Advanced Imaging, Faculty of Arts and Sciences, Harvard University, Cambridge, USA
[2]Department of Electronic and Telecommunication Engineering, University of Moratuwa, Sri Lanka
[3]Department of Mechanical Engineering, Massachusetts Institute of Technology, 77 Massachusetts Ave., Cambridge, MA 02139, USA.
[4]Laser Biomedical Research Center, Massachusetts Institute of Technology, 77 Massachusetts Ave., Cambridge, MA 02139, USA.
[5]Department of Biological Engineering, Massachusetts Institute of Technology, 77 Massachusetts Ave., Cambridge, MA 02139, USA.
[6]Picower Institute for Learning and Memory, Massachusetts Institute of Technology, 77 Massachusetts Ave., Cambridge, MA 02139, USA.
[7]Department of Neuroscience, Cleveland Clinic Lerner Research Institute, Cleveland, OH 44195, USA.
[†]equally contributed.
[*]wadduwage@fas.harvard.edu

DEEP^2 performance with different loss functions We trained the DEEP^2 inverse model with various loss functions, including MSE, KL divergence, RMSLE, and smooth L1 loss (see methods section for details) in order to identify the appropriate loss function for our reconstruction/optimization task. The performance of DEEP^2 inverse models for different loss functions on validation-synthetic-beads data, validation-mouse-pyramidal-neuronal data, validation-mouse-cortical-vascular data, and synthetic-vascular data are shown in Fig. S1, Fig. S2, Fig. S3 and Fig. S4 respectively.

We used MSE, PSNR (Peak signal-to-noise ratio), and SSIM (structural similarity index measure) for quantitative evaluation. The DEEP^2 models trained using MSE, RMSLE, and smooth L1 loss functions performed comparatively better than KL divergence in simulated test data in terms of the quantitative metrics. The DEEP^2 reconstruction for each model variation achieved qualitatively similar performance in reconstructing simulated test data. This also can be observed with line intensity plots shown. However, our the models' performance on the experimental data (as shown in Fig. S5 and Fig. S6) clearly shows that DEEP^2 inverse models trained using KL divergence loss achieved better reconstructions than other loss functions. (see B5 in Fig. S5 and C5 in Fig. S6). Therefore, we utilized KL divergence loss as the most suitable loss function to train our models to generalize toward real-world experimental data.

DEEP^2 performance with vanilla UNet In our work, we used UNet architecture with a spatial and channel squeeze and excitation (scSE) block. We trained both the vanilla UNet and scSE-UNet with KL divergence loss function to evaluate the efficiency of the scSE block for our reconstruction task. The DEEP^2 inverse model's performance for both model architectures on validation-synthetic-beads data, validation-mouse-pyramidal-neuronal data, and validation mouse-cortical-vascular data are shown in Fig. S1, Fig. S2, and Fig. S3 respectively. Both model architectures produced equally good qualitative results; however, according to the quantitative metrics, the scSE-UNet outperformed the vanilla UNet. Moreover, as shown in Fig. S5 and Fig. S6, scSE-UNet produced better reconstruction predictions for experimental data compared to the vanilla UNet. We believe including the scSE block improves the inverse model's ability to learn features effectively, thus achieving better performance than the vanilla UNet.

Generalizability of DEEP^2 An ablation study was conducted to analyze the generalizing capabilities of the DEEP^2 inverse model to different scattering lengths and datasets. The simulated cortical vasculature was utilized to study the model's generalizability across different scattering lengths. Four models were trained separately on 2, 4, and 6 scattering lengths and a dataset with mixed scattering lengths. These models were then tested on unseen test data, and the results are presented in Fig.S7. Quantitative results in Fig.S7 A, B, and C indicate that the model trained on mixed data achieved comparable performance to the best-performing model trained at its corresponding scattering length. This suggests that a single model trained on a mix of scattering length settings may generalize towards different scattering lengths if the training dataset represents all scattering lengths needed during inference.

Furthermore, the generalizability of the model trained on simulated data at 4 scattering lengths from a mixture of data distributions with mouse-cortical-vasculature data, mouse-pyramidal-neuronal data, and synthetic-beads data was studied. The quantitative results presented in Fig.S8 A, B, and C show that the model trained on mixed training data failed to generalize. The model trained on mixed data performed better with the mouse pyramidal neurons than the regular model trained only on mouse pyramidal neuronal data. In contrast, the mixed-trained model performed poorly with the synthetic beads and mouse cortical vasculature. This indicates that it is necessary to train separate models for different datasets to obtain accurate reconstructions. We did not observe a noticeable qualitative difference between the model trained on mixed data and the regular-trained model for experimental cortical vasculature, as seen in Fig.S8E. Note that for this study, the training data was simulated using the forward model settings (i.e., the patterns, scattering PSF, etc.) of cortical vasculature data at 4 scattering lengths. Therefore, we only tested experimental data for cortical vasculature.

Using transfer learning, we also evaluated the inverse models' generalizability and robustness to out-of-distribution datasets. We trained 3 separate models on mouse-cortical-vasculature, mouse-pyramidal-neuron, and bead datasets at 4 scattering lengths. Then we tested them on all three datasets (i.e., one in-distribution dataset, and two out-distribution datasets). The results are shown in Fig.S15. The model pretrained on mouse cortical vasculature, and the model pretrained on mouse pyramidal neurons performed comparably on out-of-distribution test data even without any target-dataset-specific finetuning. But the model pre-trained on the bead dataset didn't generalize to the other two datasets. We also evaluated the transfer-learning performance of the model pretrained on the mouse pyramidal neuron dataset. We individually fine-tuned it on the bead dataset as well as on the mouse cortical vasculature dataset for 20 epochs. Both the fined-tuned models performed well on their corresponding target datasets. As shown in Fig.S15 the quality of reconstruction improved after finetuning. This clearly indicates that our inverse model generalizes to out-of-distribution data in a transfer learning setting.

Number of patterns vs. performance of DEEP^2 Our work shows that the DEEP^2 inverse model can reconstruct a de-scattered image from stacks of DEEP measurements. We trained our model with 1, 2, 4, 8, 16, and 32 patterned measurements to show the relationship between the number of patterns and the performance of the inverse model. Fig.S9 shows the effect of the number of patterns on DEEP^2 reconstruction on the mouse cortical vasculature dataset. The MSE (lower the better) decreased monotonically as the number of patterns were increased. Similarly, the PSNR (higher the better) and SSIM (higher the better) increased monotonically. We believe that the higher number of patterns could give better performance, but there was a tradeoff between performance and computational requirements. As shown in Fig.S9E for the 2 scattering lengths mouse cortical vasculature data, a stable and satisfactory performance was achieved even with 4-patterns, but 6 scattering lengths mouse cortical vasculature data required a minimum of 32 patterns to yield a satisfactory performance. In conclusion, we chose 32 as the number of patterns considering the computational limitations and the model's performance with different numbers of patterns.

Sensitivity analysis on DEEP^2 We conducted two experiments to evaluate the sensitivity of the DEEP^2 inverse model. First, we trained a model on 2 scattering lengths of mouse cortical vasculature data and tested its performance on 1.6 to 2.4 scattering lengths. Second, we trained a model on a mixture of 2, 4, and 6 scattering lengths of mouse cortical vasculature data and tested it on 1.6 to 2.4 scattering lengths data. As shown in Fig. S10 the models' performance was optimum when the train data matched the validation data for both experiments. The lowest MSE and highest SSIM and PSNR were observed at 2 scattering lengths, and the performance gradually decreased as the validation data deviated from train data. This decrease was lower for the model trained on a mixture of scattering lengths. This suggests that the DEEP^2 model may generalize to a range of depth conditions if the training data represents all the depth conditions. We did not observe a noticeable qualitative difference in the reconstructed images (see Fig. S10D).

Ablation study on the forward model We conducted an ablation study on the forward model by removing different components: the scattering model, EMCCD noise model, and the electron multiplication noise (EM-noise) model. For each of these ablations, a simulated training dataset of cortical vasculature at 2 scattering lengths was generated. Representative images from each ablation are shown in Fig. S11A. Then each training dataset was used to train a separate inverse model. All models converged (see the quantitative numerical test performance in the solid black bars in Fig. S11B-D.). Then we tested the trained models on numerical test data generated from the original forward model (without ablations). As seen in Fig. S11B-D, removing EM-noise from the forward model minimally affected the inverse model performance. But removing the entire EMCCD noise model, and removing the scattering model rapidly degraded the performance. We also show representative qualitative results in Fig. S11E. Finally, each inverse model trained on ablated datasets was tested on experimental cortical vasculature data. As seen in Fig. S11F all trends seen in the numerical validations were present on the experimental test results. This study shows the importance of our scattering and EMCCD noise models.

Number of patterns vs. performance of DEEP To establish a baseline for DEEP^2's performance, we evaluated the performance of the DEEP-TFM mathematical inverse algorithm (without regularization) by varying the number of patterns

used to reconstruct. The performance of DEEP was evaluated on both simulated mouse cortical vasculature data (See Fig.S12) and experimental data (See Fig.S13). The quality of reconstruction by the DEEP algorithm on the 2 scattering lengths mouse cortical vasculature dataset increased with the number of patterns, such increment was reflected in the quantitative metrics - MSE, and PSNR. According to Fig.S12 the DEEP reconstruction algorithm failed to reconstruct 4 and 6 scattering lengths data with 32 patterns. A similar observation can be seen with the experimental data (shown in Fig.S13). This indicates that the DEEP algorithm requires more patterns to reconstruct data at higher scattering lengths. The performance of the DEEP algorithm on experimental data was further evaluated by varying the number of patterns ($2-255$). The quantitative metrics were calculated by considering the reconstruction from 255 patterns as the pseudo-ground truth. As shown in Fig.S13A-D the performance of the DEEP algorithm on experimental data increased with the number of patterns. 2, 4, and 6 scattering lengths mouse cortical vasculature data required a minimum of 32, 48, and 235 patterns to achieve acceptable reconstruction.

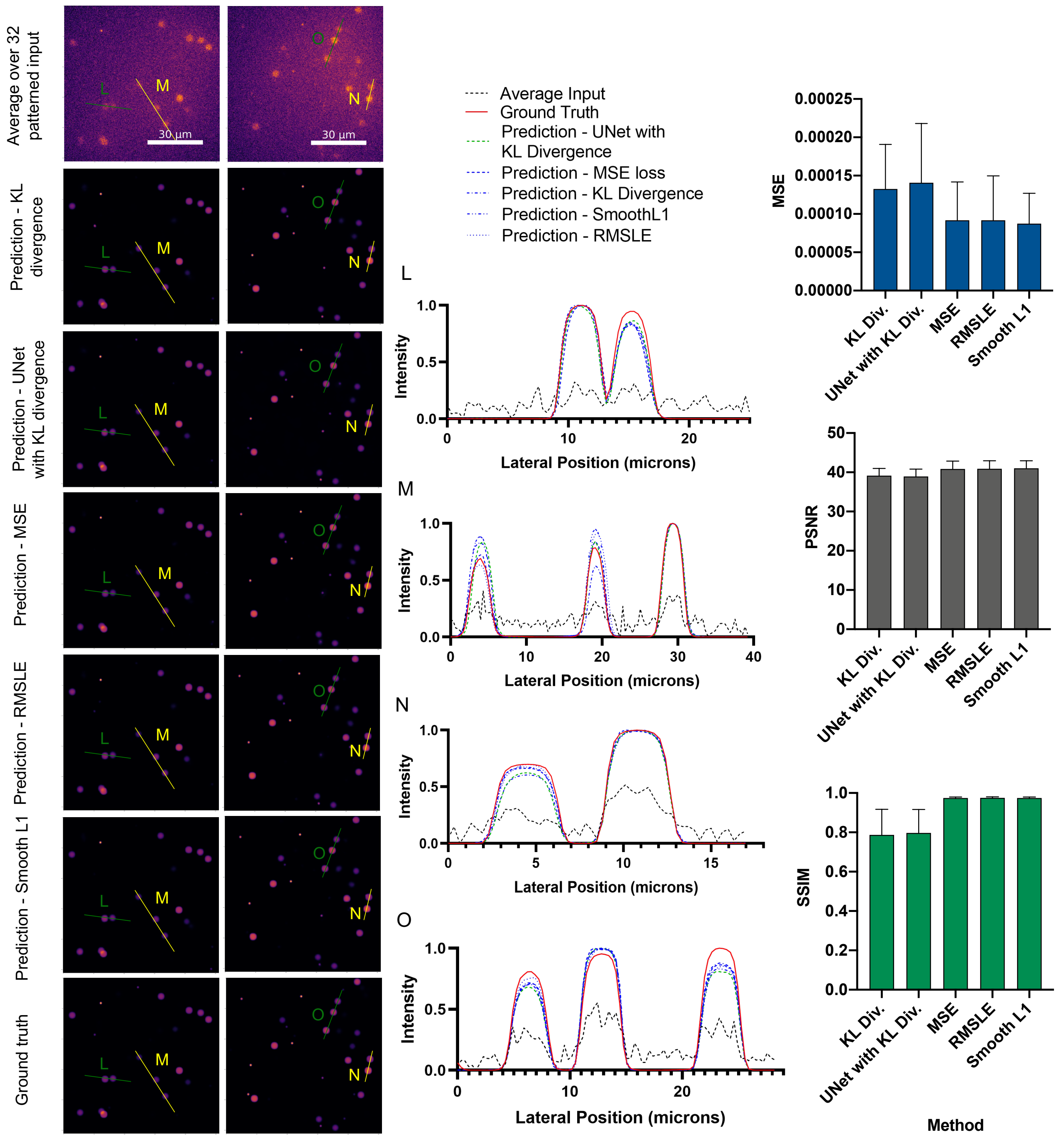

Figure S1. Performance comparison of variations of the DEEP^2 inverse model for validation synthetic beads data at 4 scattering lengths below the surface. Five model variations were compared: (1) scSE-Unet with smooth-L1 loss, (2) scSE-Unet with RMSLE loss, (3) scSE-Unet with MSE loss, (4) scSE-Unet with KL-divergence loss, and (5) vanilla-Unet with KL-divergence loss. Note that the model variations in the figure are labeled with the loss function used. Unless mentioned, the model architecture is the scSE-Unet (results from vanilla Unet are labeled as Unet with KL-Divergence). DEEP^2 reconstructions for each model variation, along with the simulated DEEP-TFM image stacks (averaged over the 32 patterns), and ground truth images are shown on the left. The intensity along the yellow lines L, M, N, and O are visualized in the plots (L), (M),(N), and (O) shown in the middle. Quantitative evaluation plots –i.e. mean squared error (MSE), peak signal-to-noise ratio (PSNR), and structural similarity index measure (SSIM)– for each model variation are shown on the right. The error bars indicate the standard deviation.

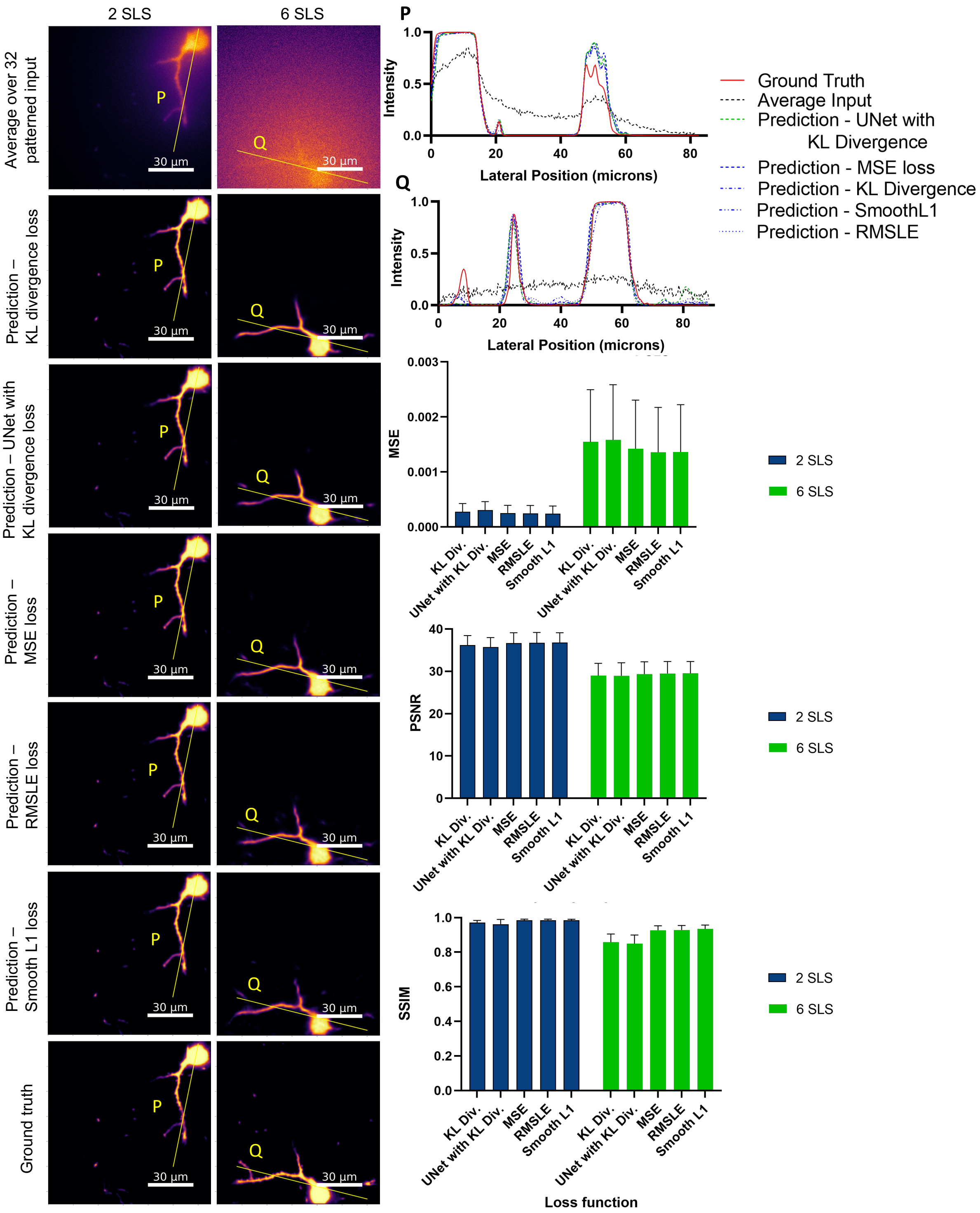

Figure S2. Performance comparison of variations of the DEEP^2 inverse model for validation-mouse-pyramidal-neuron data at 2 and 6 scattering lengths (SLS) below the surface. Five model variations were compared: (1) scSE-Unet with smooth-L1 loss, (2) scSE-Unet with RMSLE loss, (3) scSE-Unet with MSE loss, (4) scSE-Unet with KL-divergence loss, and (5) vanilla-Unet with KL-divergence loss. Note that the model variations in the figure are labeled with the loss function used. Unless mentioned, the model architecture is the scSE-Unet (results from vanilla Unet are labeled as Unet with KL-Divergence). DEEP^2 reconstructions for each model variation, along with the simulated DEEP-TFM image stacks (averaged over the 32 patterns), and ground truth images are shown on the left. The intensity along the yellow lines P, and Q are visualized in the plots (P), and (Q) shown on the top right. Quantitative evaluation plots –i.e., mean squared error (MSE), peak signal-to-noise ratio (PSNR), and structural similarity index measure (SSIM)– for each model variation are shown on the bottom right. The error bars indicate the standard deviation.

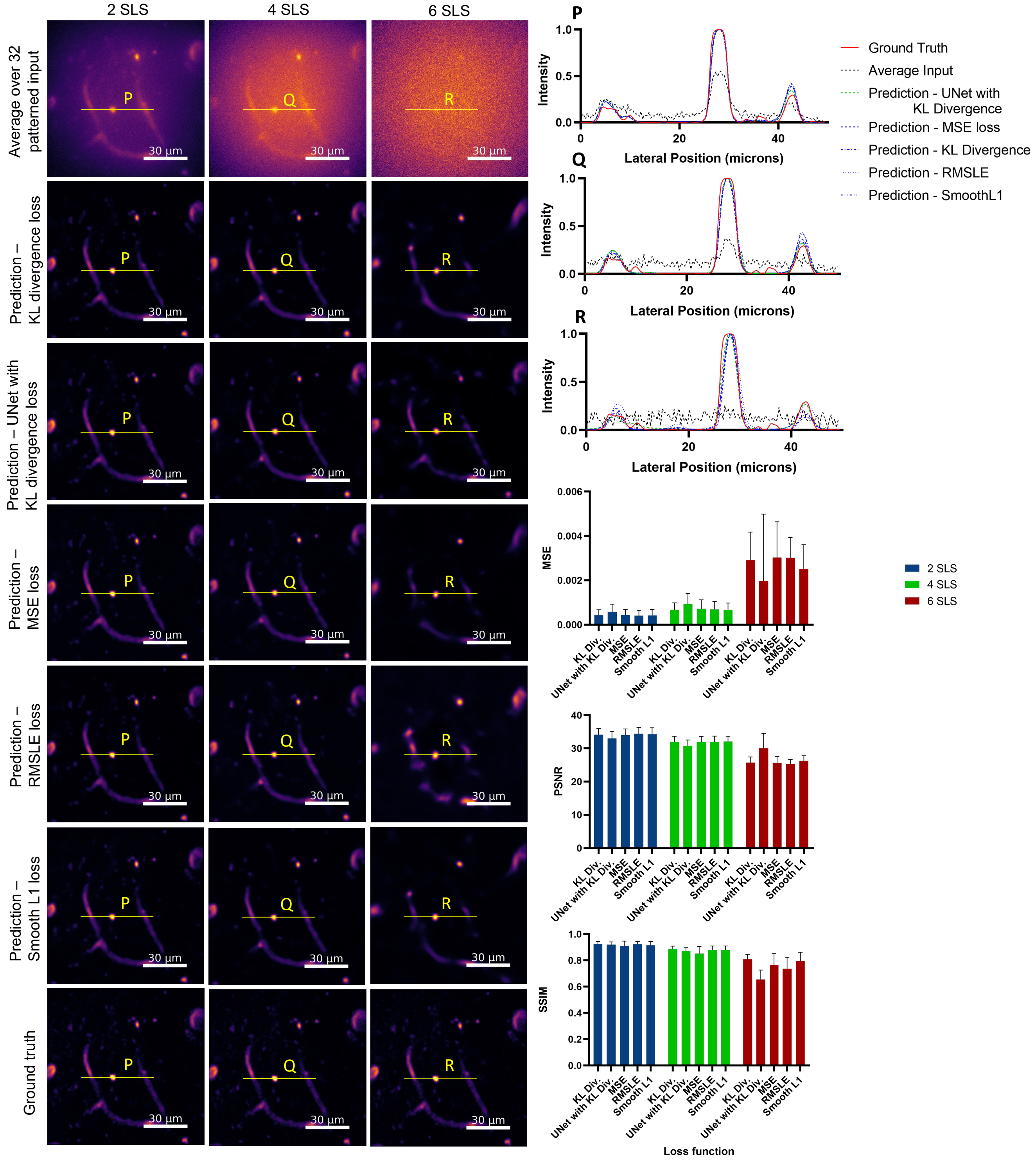

Figure S3. Performance comparison of variations of the DEEP^2 inverse model for validation-mouse-cortical-vasculature data at 2, 4, and 6 scattering lengths (SLS) below the surface. Five model variations were compared: (1) scSE-Unet with smooth-L1 loss, (2) scSE-Unet with RMSLE loss, (3) scSE-Unet with MSE loss, (4) scSE-Unet with KL-divergence loss, and (5) vanilla-Unet with KL-divergence loss. Note that the model variations in the figure are labeled with the loss function used. Unless mentioned, the model architecture is the scSE-Unet (results from vanilla Unet are labeled as Unet with KL-Divergence). DEEP^2 reconstructions for each model variation, along with the simulated DEEP-TFM image stacks (averaged over the 32 patterns), and ground truth images are shown on the left. The intensity along the yellow lines P, Q and R are visualized in the plots (P), (Q) and (R) shown on top right. Quantitative evaluation plots –i.e., mean squared error (MSE), peak signal-to-noise ratio (PSNR), and structural similarity index measure (SSIM)– for each model variation are shown on the bottom right. The error bars indicate the standard deviation.

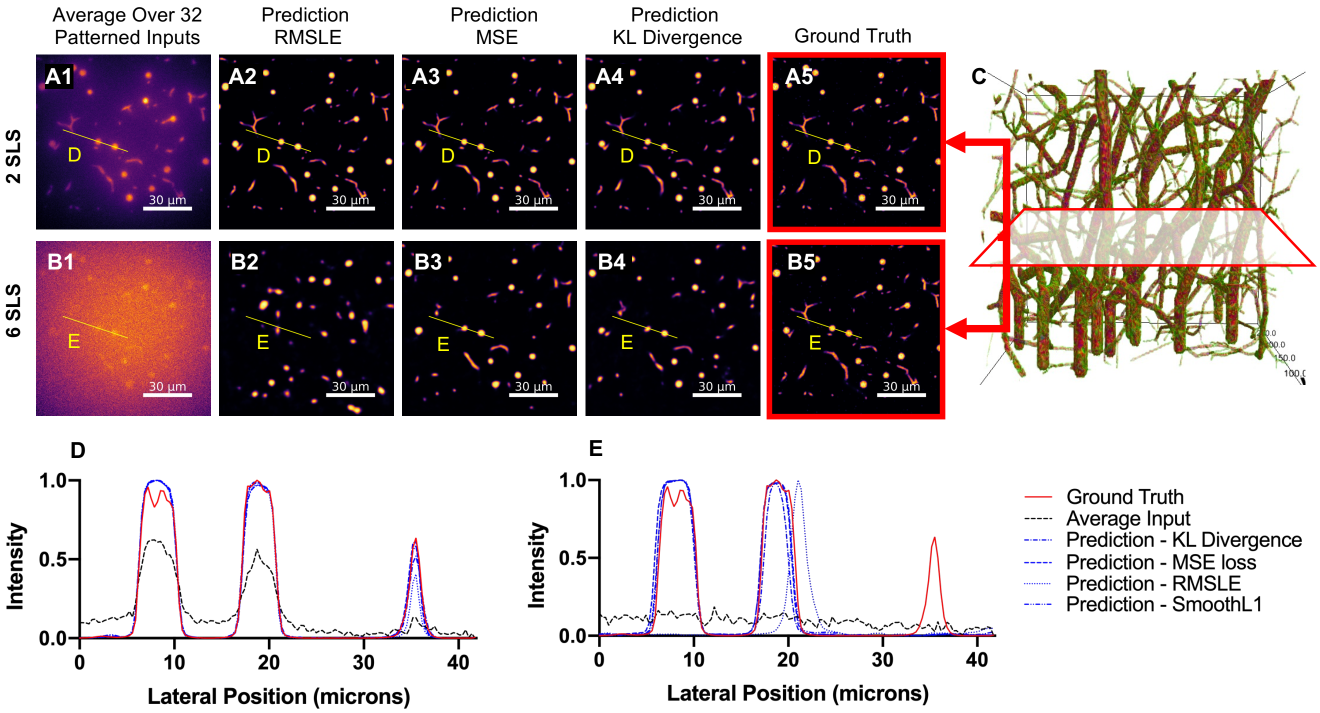

Figure S4. Additional verification of the DEEP^2 reconstruction on synthetic vasculature object (C) at 2, and 6 scattering lengths (SLS) below the surface. **(A1) & (B1)** Simulated DEEP-TFM image stacks (averaged over the 32 patterns) at 2 and 6 scattering lengths, respectively. **(A1-4) & (B1-4)** DEEP^2 reconstructions by scSE-Unet with different loss functions. **(A5) & (B5)** Ground truth images corresponding to (A1) & (B1). The intensity along the yellow lines E, and E are visualized in the plots (D), and (E).

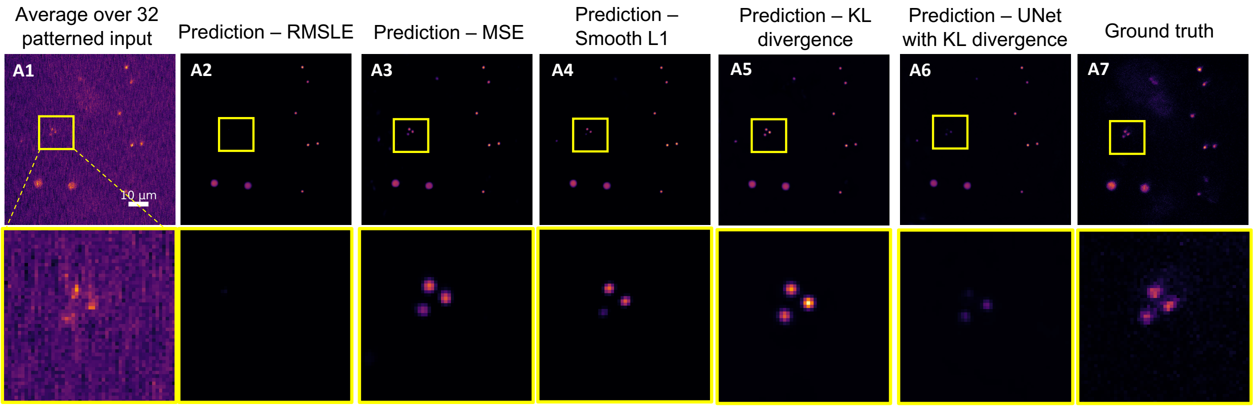

Figure S5. Performance comparison of variations of the DEEP^2 inverse model for experimental beads data at 4 scattering lengths below the surface. Five model variations were compared: (1) scSE-Unet with smooth-L1 loss, (2) scSE-Unet with RMSLE loss, (3) scSE-Unet with MSE loss, (4) scSE-Unet with KL-divergence loss, and (5) vanilla-Unet with KL-divergence loss. Note that the model variations in the figure are labeled with the loss function used. Unless mentioned, the model architecture is the scSE-Unet (results from vanilla Unet are labeled as Unet with KL-Divergence). DEEP^2 reconstructions for each model variation (A2-A6), along with the simulated DEEP-TFM image stacks (averaged over the 32 patterns) (A1), and ground truth images (A7) are shown. The yellow colored boxes on (A1-A7) images are enlarged for close visualization in (B1-B7). Visual observations suggest that scSE-Unet with KL-divergence loss worked best.

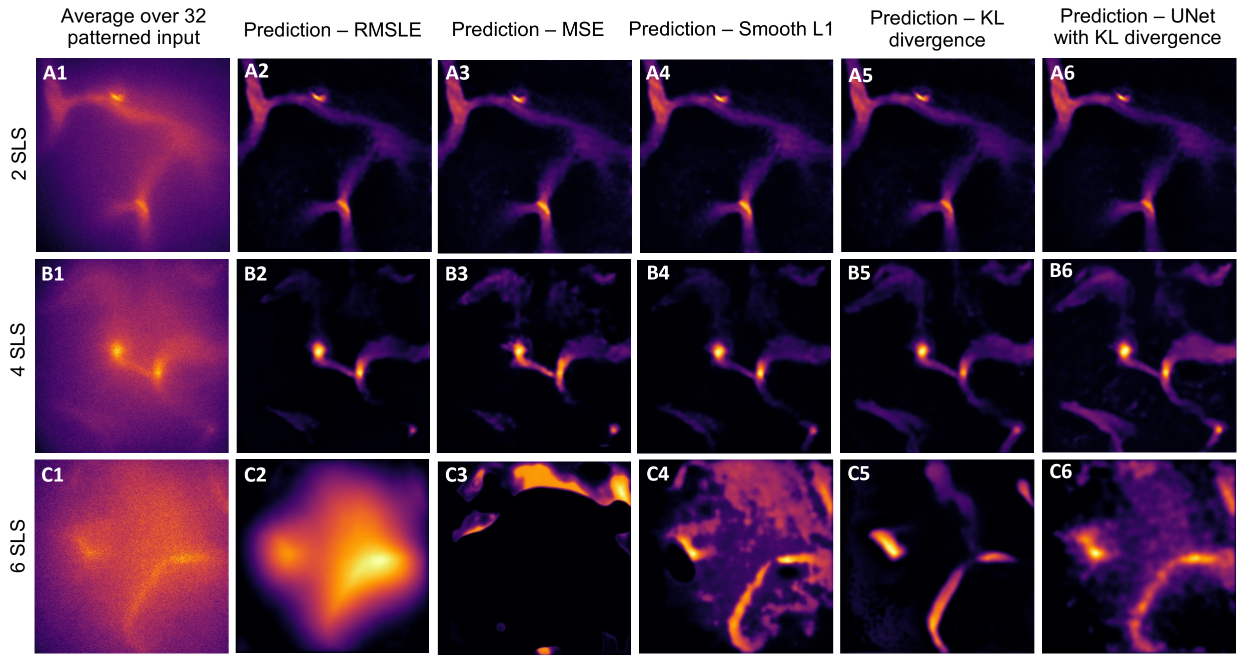

Figure S6. Performance comparison of variations of the DEEP^2 inverse model for experimental mouse cortical vasculature data at 2, 4, and 6 scattering lengths (SLS) below the surface. Five model variations were compared: (1) scSE-Unet with smooth-L1 loss, (2) scSE-Unet with RMSLE loss, (3) scSE-Unet with MSE loss, (4) scSE-Unet with KL-divergence loss, and (5) vanilla-Unet with KL-divergence loss. Note that the model variations in the figure are labeled with the loss function used. Unless mentioned, the model architecture is the scSE-Unet (results from vanilla Unet are labeled as Unet with KL-Divergence). DEEP^2 reconstructions for each model variation (A2-A6), along with the simulated DEEP-TFM image stacks (averaged over the 32 patterns) (A1) are shown. Visual observations suggest that scSE-Unet with KL-divergence loss worked best (see C5).

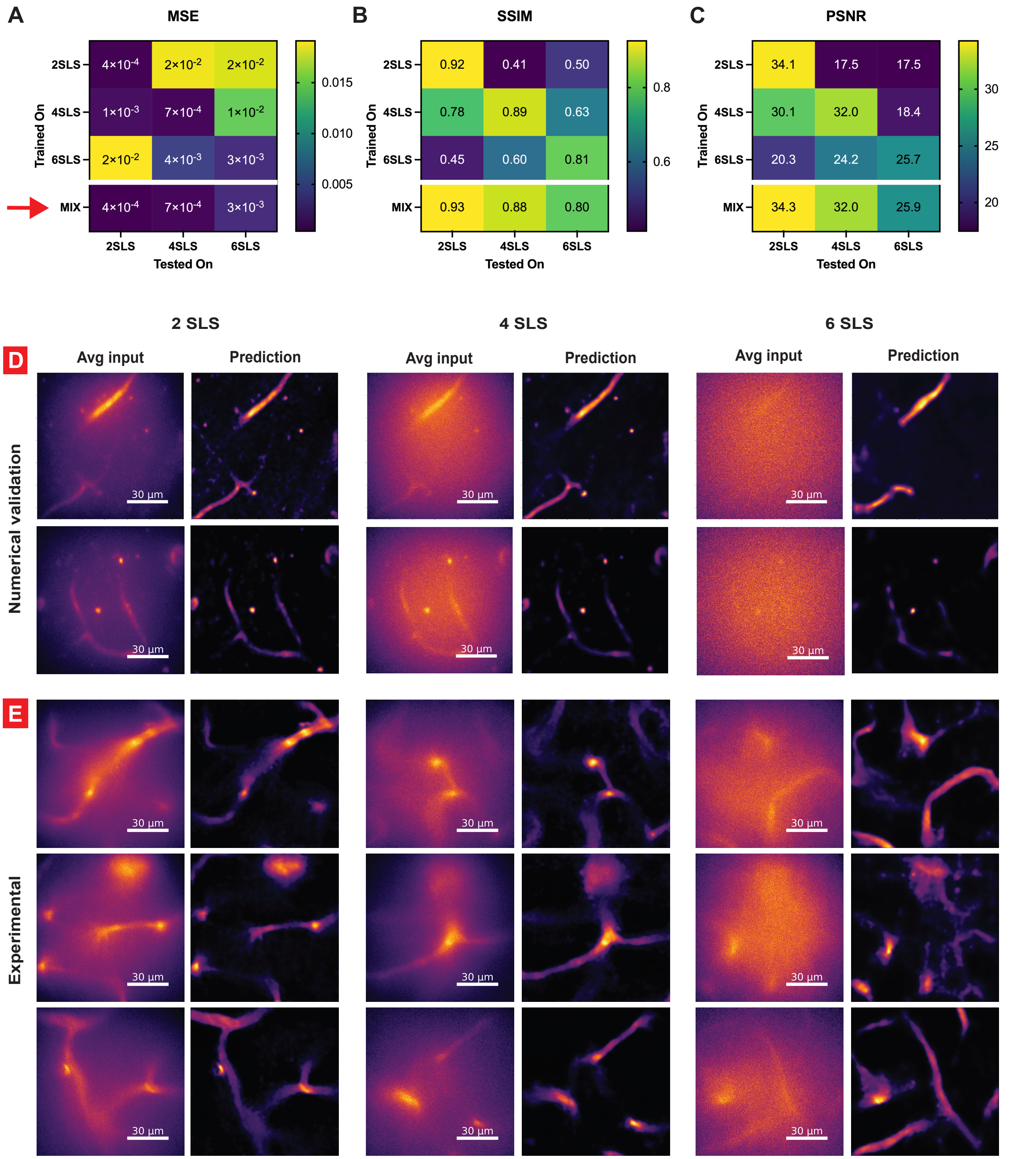

Figure S7. Generalizability of models trained on simulated data of cortical vasculature at a particular scattering length vs. a model trained on a mix of scattering lengths. Three models, each trained on 2, 4, and 6 scattering lengths (SLS) were tested on unseen numerical data from all 2, 4, and 6 scattering lengths. Another model was trained on a dataset with a mix of 2, 4, and 6 scattering lengths and tested the same way. (A-C) Confusion matrices show the performance of all four models on all three scattering lengths. (A) Mean squared error (MSE). (B) Structural similarity index measure (SSIM). (C) Peak signal-to-noise ratio (PSNR). Note that the model trained on the mix performed comparable to the best-performing model trained on its corresponding scattering length (see the red arrow). (D-E) Qualitative results for the model trained on the mix of scattering lengths. In both numerical validations and experimental tests, the model generalized to all three conditions.

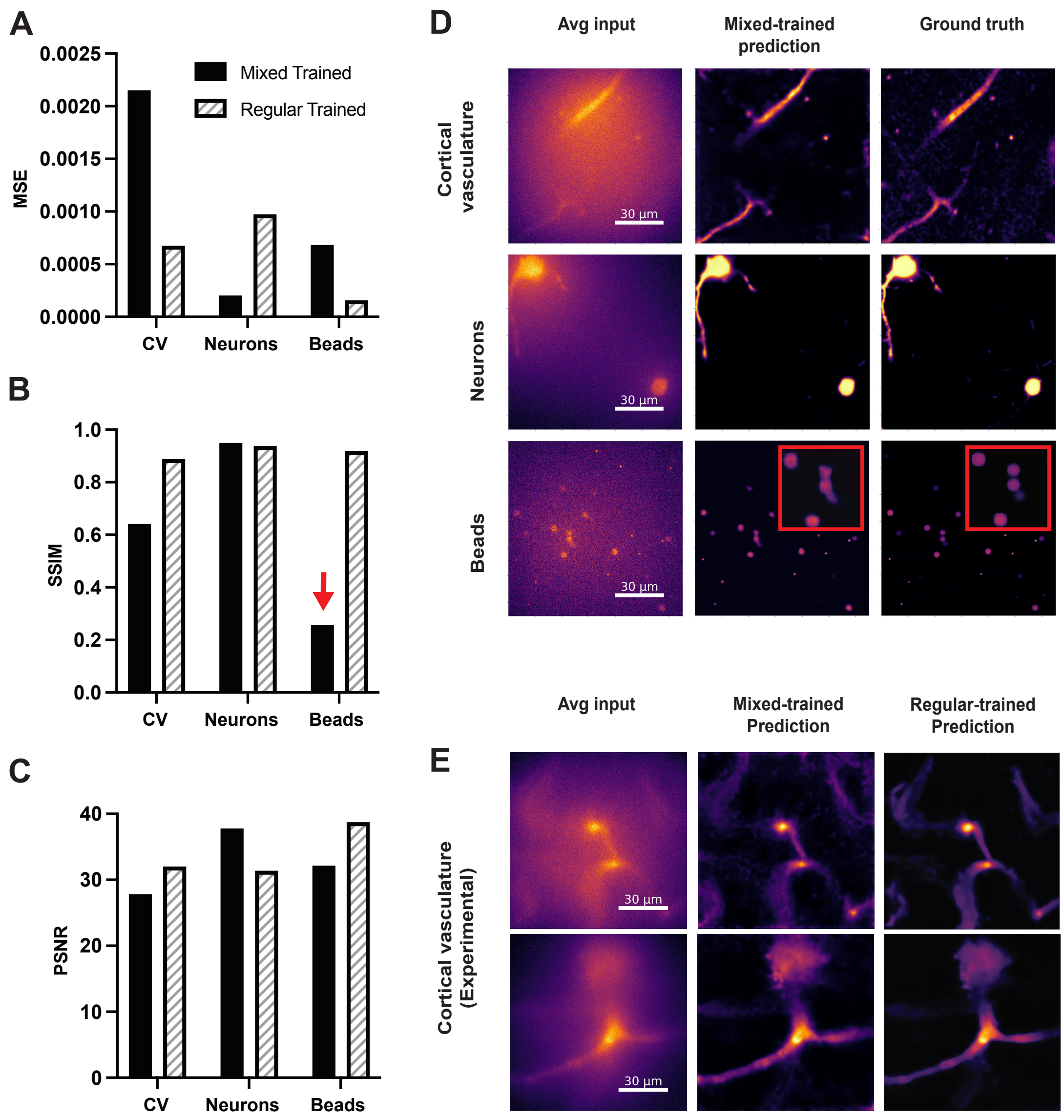

Figure S8. Generalizability of an inverse model trained on simulated data at 4 scattering lengths from a mix of data distributions with three types of images (cortical vasculature, neurons, and artificial fluorescent beads). (A-C) Quantitative performance. In the "regular trained column," we compare the best-performing model trained on the correct data distribution. (D) Qualitative results on numerical validations. (E) Qualitative results on the cortical vasculature experimental test dataset at 4 scattering length. In general, model performance decreased when trained on a mix of data distributions. Especially for the beads dataset, there was a significant performance decrease (see the red arrow in 'B' and red insets in 'D').

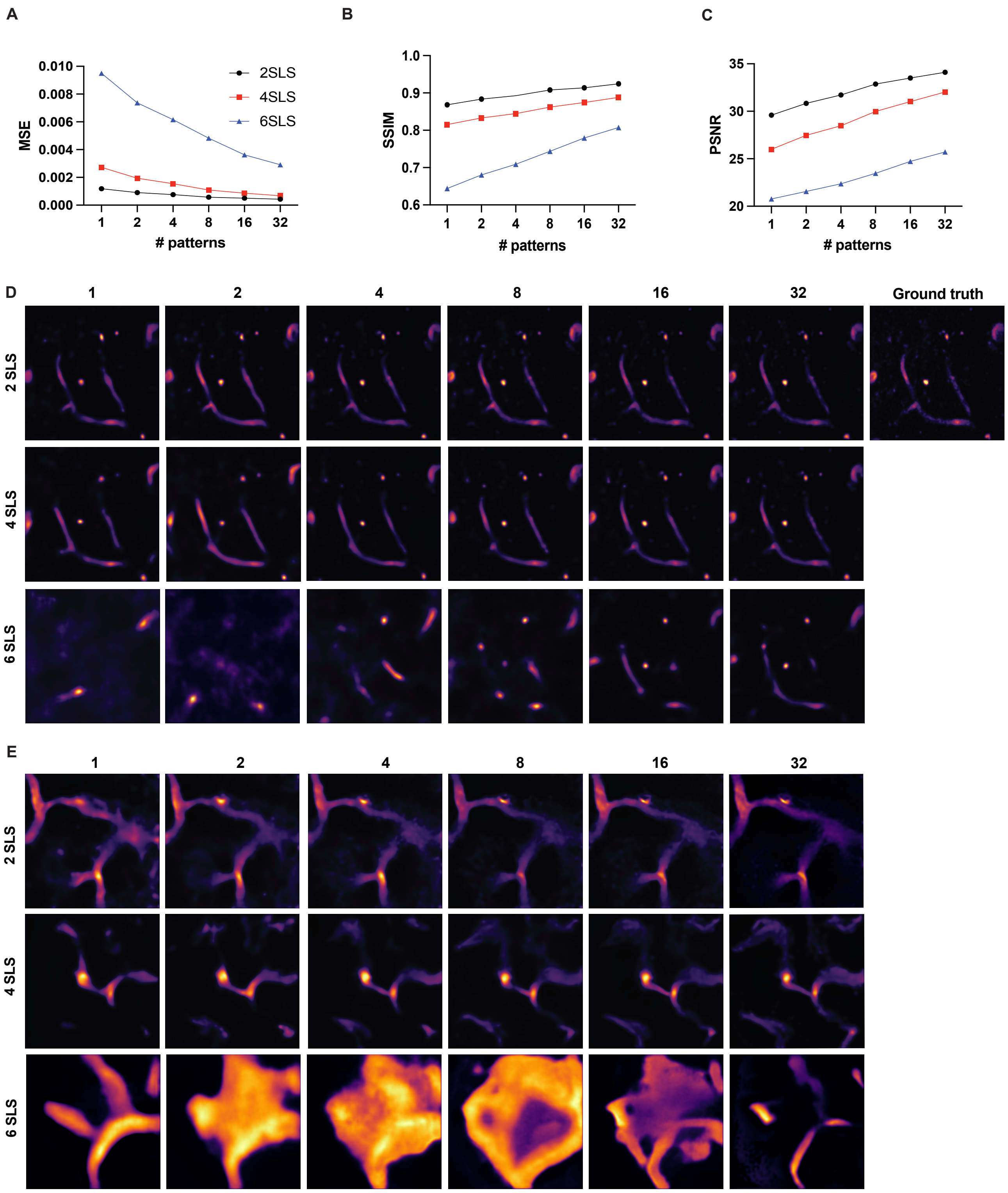

Figure S9. Effect of the number of patterns on DEEP^2 reconstruction. Inverse models that reconstruct from 1, 2, 4, 8, 16, and 32 patterned excitations were trained on simulated cortical vasculature data from 2, 4, and 6 scattering lengths (SLS). In total, 18 (=6x3) models were trained. (A) Mean squared error (MSE) vs. number of excitation patterns. (B) Structural similarity index measure (SSIM) vs. number of excitation patterns. (C) Peak signal-to-noise ratio (PSNR) vs. number of excitation patterns. (D). Qualitative results on numerical validations. (E) Qualitative results on experimental tests.

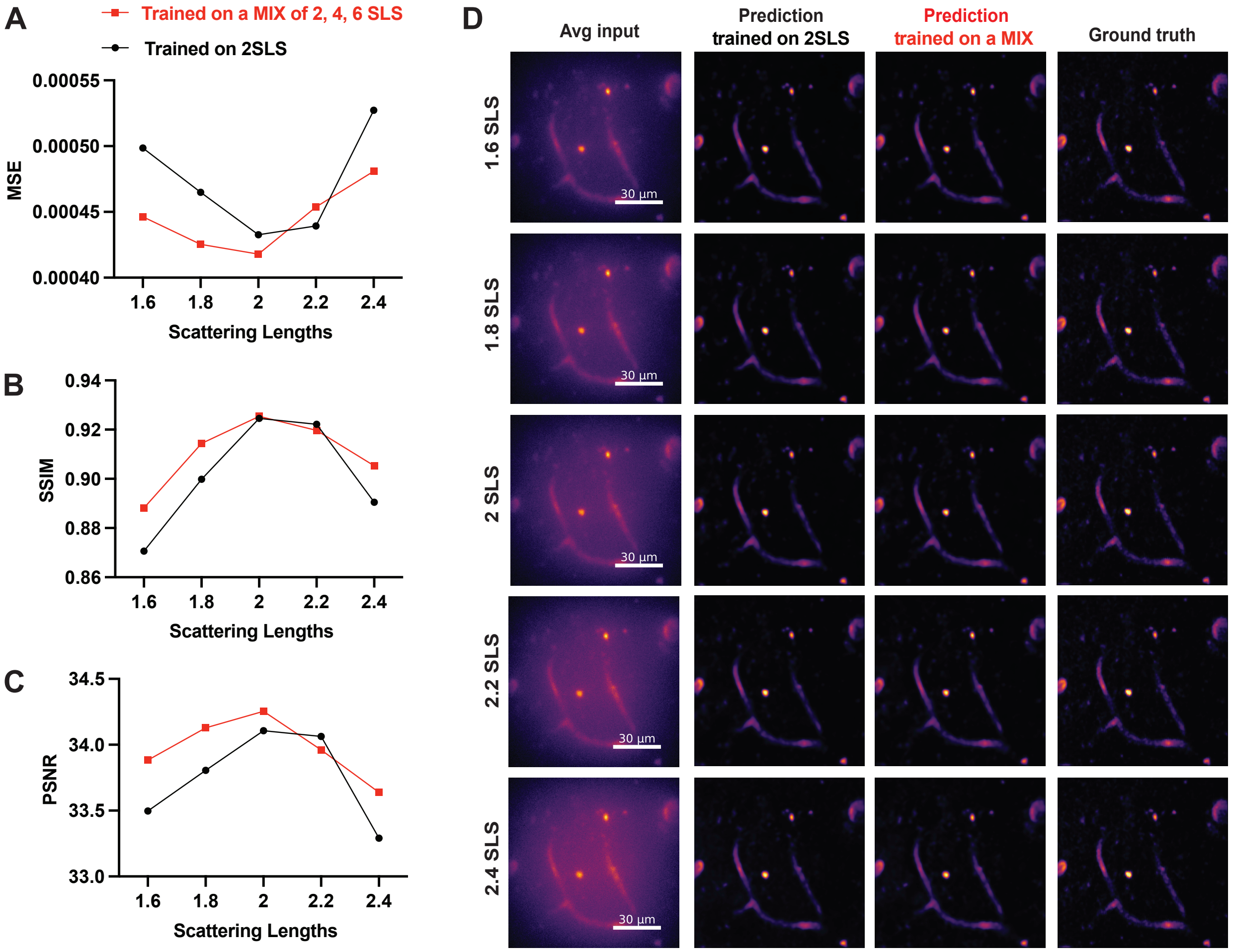

Figure S10. Sensitivity analysis on the DEEP^2 inverse model trained on simulated data of cortical vasculature at two scattering lengths. The model was tested on unseen numerical data generated at 1.6, 1.8, 2, 2.2, and 2.4 scattering lengths (SLS). In addition, the inverse model trained on simulated data at a mix of 2, 4, and 6 scattering lengths was also tested the same way (results shown in red). (A) Mean squared error (MSE) vs. scattering length. (B) Structural similarity index measure (SSIM) vs. scattering length. (C) Peak signal-to-noise ratio (PSNR) vs. scattering length. (D). Qualitative results.

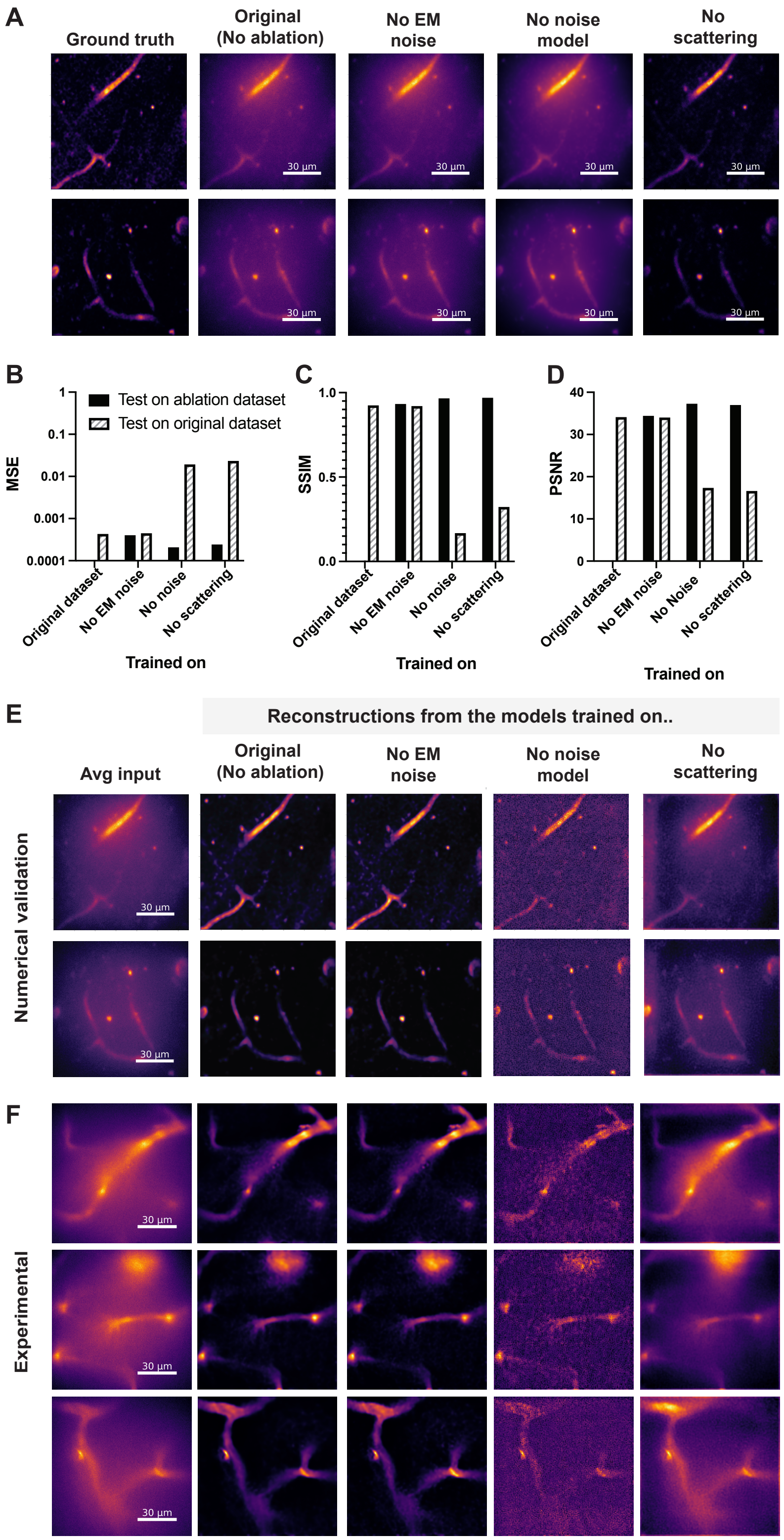

Figure S11. The ablation study on removing different components of the forward model. (A) Representative simulated images generated from ablated forward model. We tested for removing the noise added by the electron multiplication process (EM-noise), removing the entire noise model, and removing the scattering model. (B-D) Quantitative performance of the inverse models trained on ablated datasets. Please note that the "Test on original dataset" is the informative metric. (B) Mean squared error (MSE). (C) Structural similarity index measure (SSIM). (D) Peak signal-to-noise ratio (PSNR). (E-F) Qualitative results for the same ablations. Numerical validations are on the original validation data (without ablations). Both numerical validations and experimental tests show similar trends. Ablating EM-noise marginally affected the performance, while ablating the entire noise and scattering models degraded the performance.

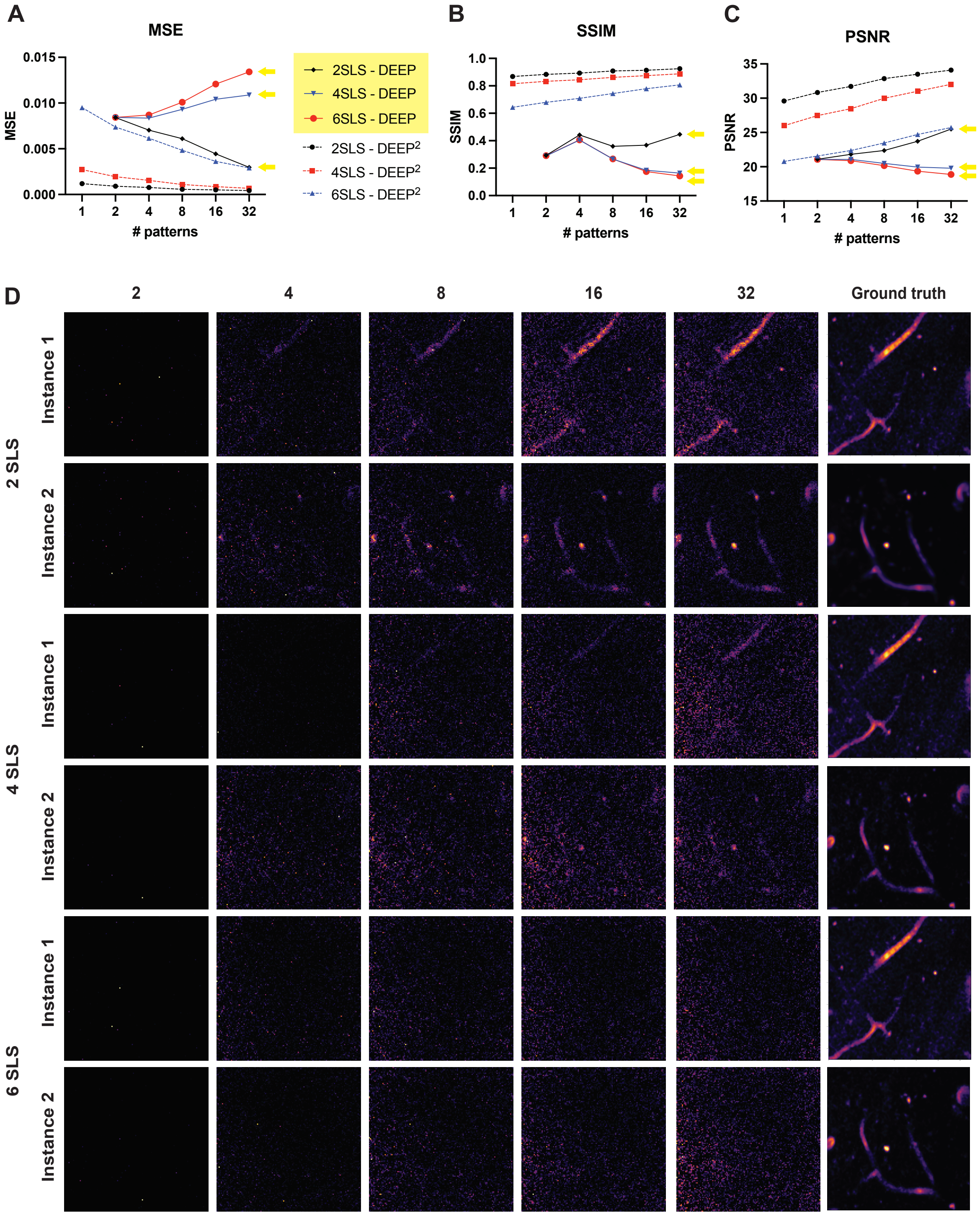

Figure S12. Evaluation of DEEP's performance for numerical validation data by varying the number of patterns used to reconstruct. (A-C) Quantitative performance metrics vs. number of patterns. The dotted lines show DEEP^2 performance under the same conditions for reference. (D) Qualitative reconstruction results on two representative validation instances. Notice that only 2 scattering length (SLS) case was able to reconstruct to a good performance, and hence the quantitative metrics improve with the increasing number of patterns. For 4 and 6 scattering lengths, quantitative metrics do not capture the reconstruction with increasing number of patterns (notice how the noise also increases with increasing patterns).

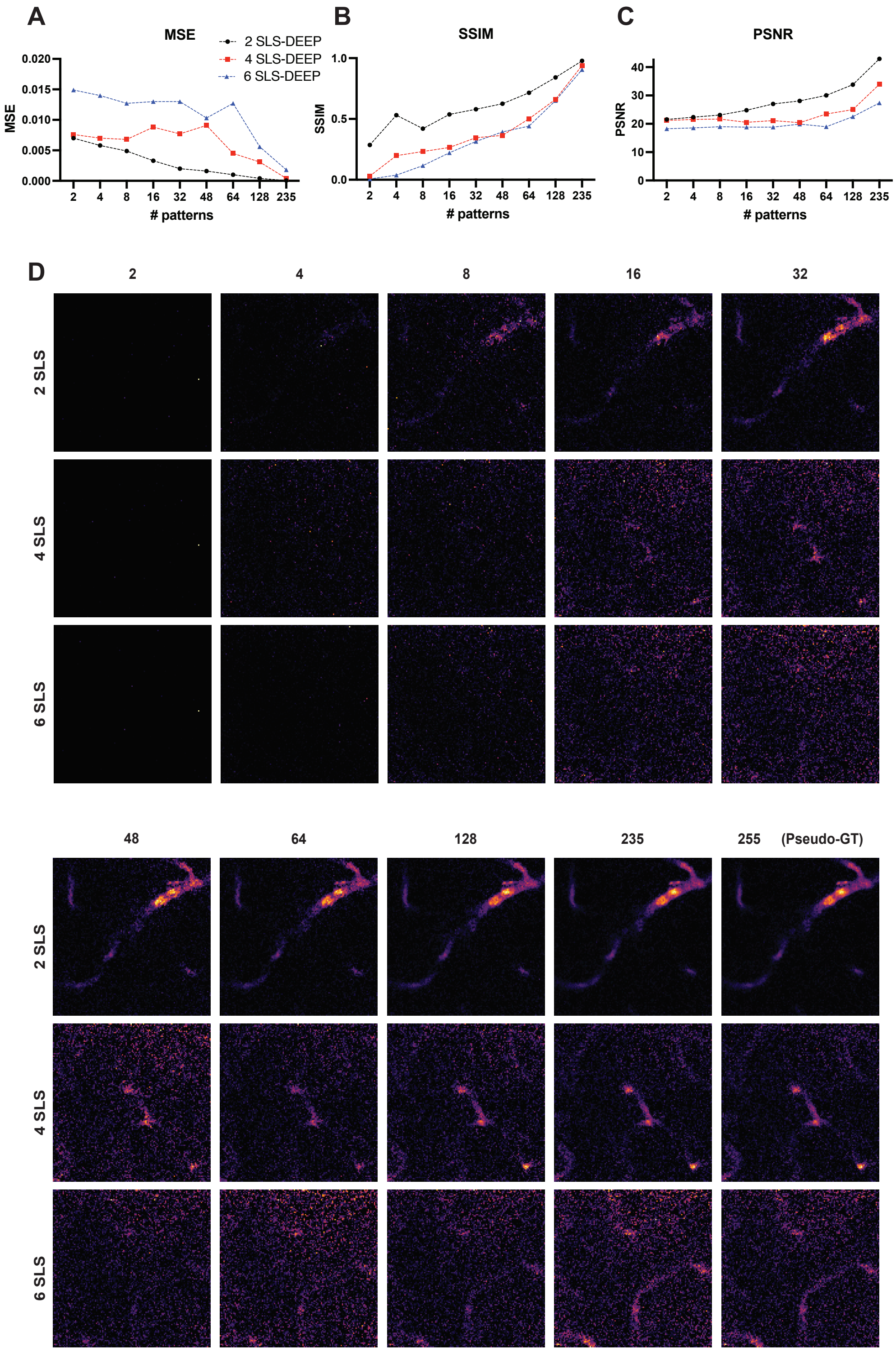

Figure S13. Evaluation of DEEP's performance for experimental cortical vasculature data by varying the number of patterns used to reconstruct. (A-C) Quantitative performance metrics vs. number of patterns. Here we treat the reconstruction from 255 patterns as the pseudo ground truth to calculate the MSE, SSIM, and PSNR. (D) Qualitative reconstruction results on a representative validation instance from each scattering length (SLS) depth. Notice that only 2 scattering length case was able to reconstruct to a good performance with 32 patterned excitations agreeing with the numerical validations. A larger number of patterns were needed for 4 and 6 scattering length depths.

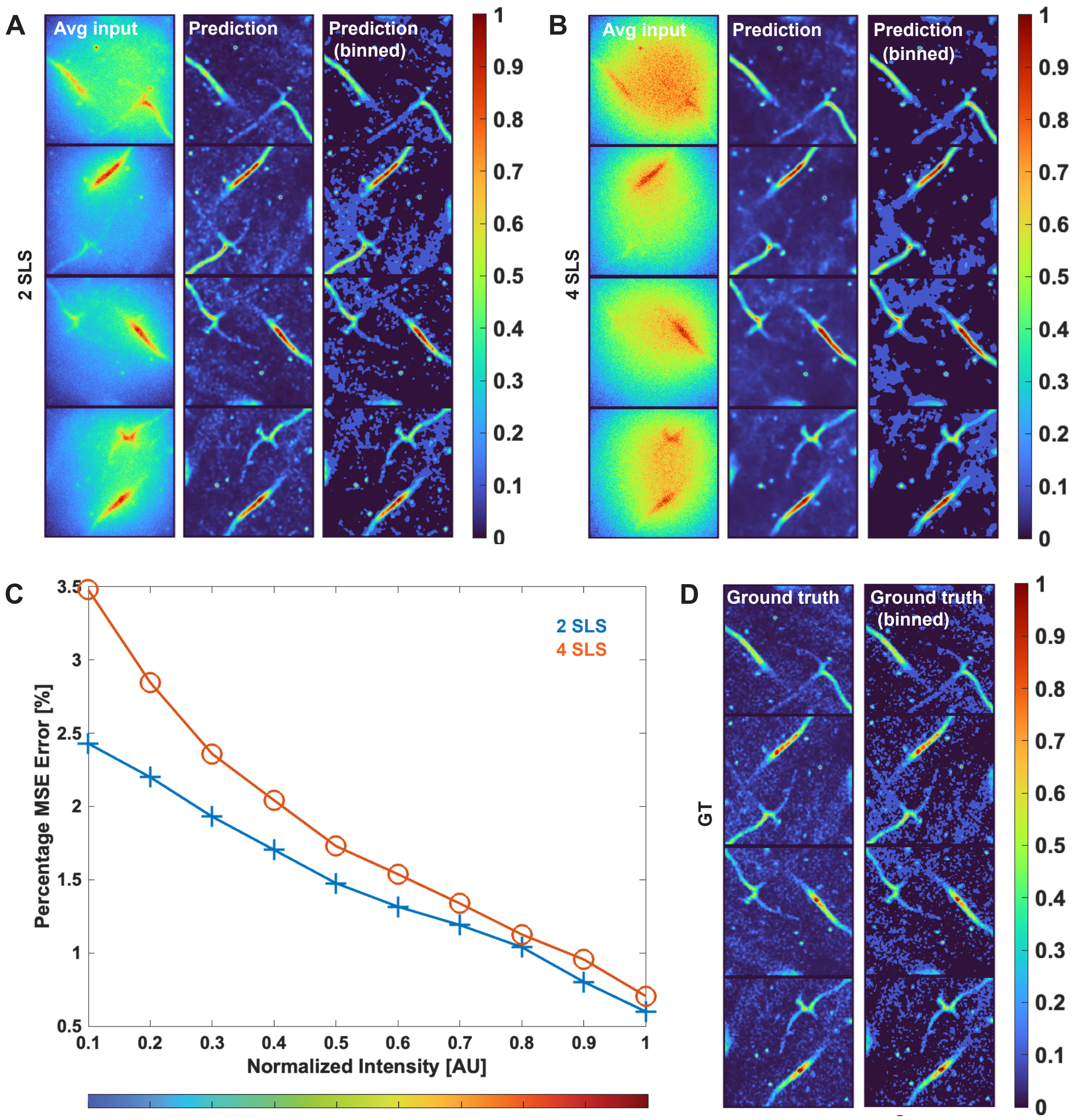

Figure S14. Analysis of error by intensity levels. Normalized intensity levels were binned into ten bins with 0.1 increments. Then the percentage error of MSE was calculated for each bin across all test images, and the average was reported. (A) The average input image, the prediction, and the binned prediction for four representative images from the test set at 2 scattering lengths (SLS). (B) The average input image, the prediction, and the binned prediction for 4 scattering lengths. (C) The percentage error in MSE for each bin. (D) The ground truth and the binned ground truth for reference.

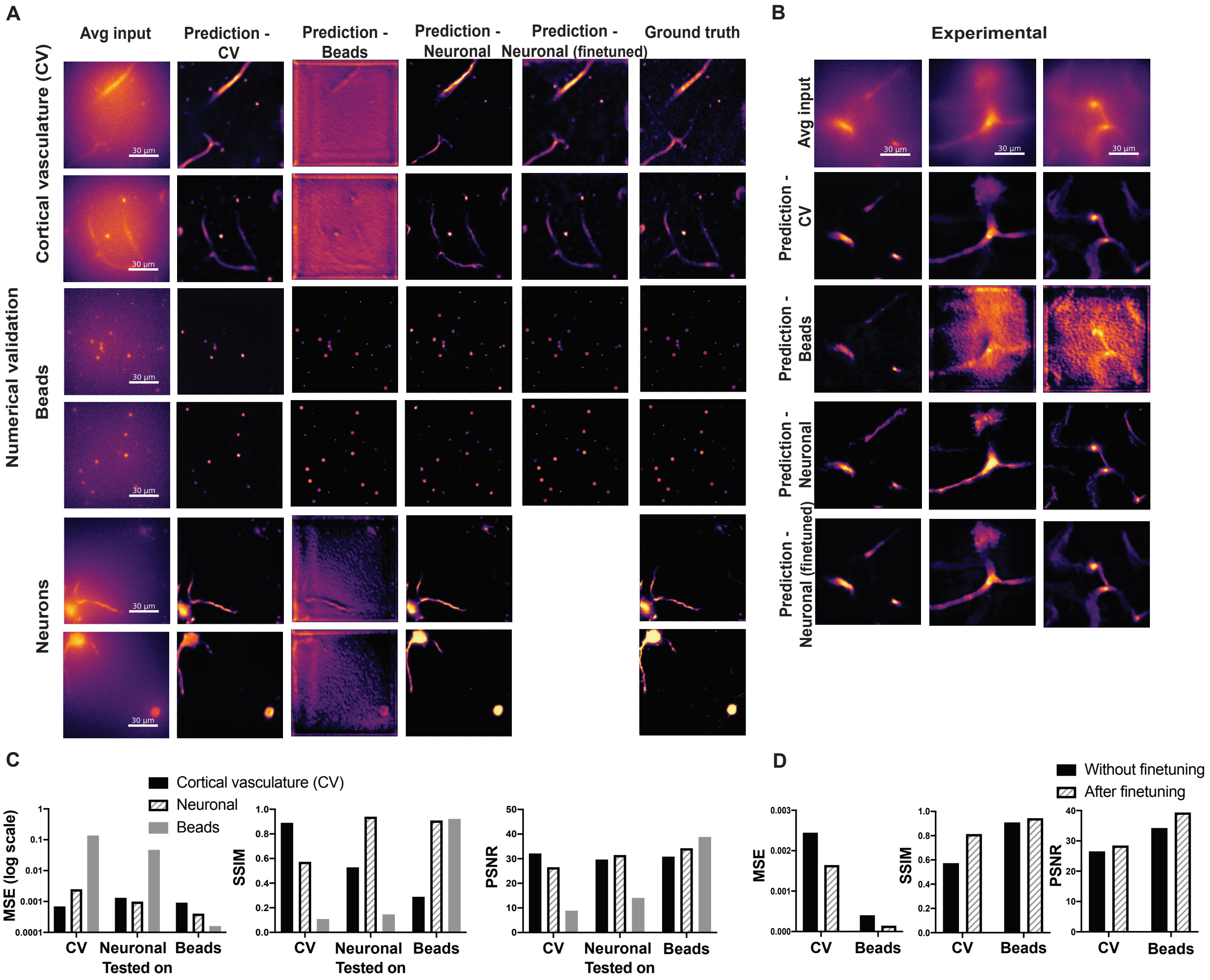

Figure S15. Generalizability and robustness of DEEP^2 inverse models trained on simulated data at 4 scattering lengths (SLS) for cortical vasculature (CV), neuronal, and beads data sets. (A) The average input image and predictions of the following models: cortical vasculature pretrained model; beads pretrained model; neuronal pretrained model without target dataset specific finetuning; and neuronal pretrained model after target dataset specific finetuning. The ground truth images are shown in the last column for reference. Two representative instances for each dataset are provided. (B) Qualitative performance evaluation of the pretrained models on the experimental cortical vasculature dataset before and after finetuning. (C) Quantitative evaluation of pretrained models on each dataset without target-specific finetuning. Note that the x-axis indicates the dataset the model was tested on, and the legend indicates the dataset the model was pretrained on. (D) Quantitative performance comparison between the neuronal pretrained model before and after finetuning on cortical vasculature and beads datasets.
